# Impact of a weight loss and fitness intervention on exercise‐associated plasma oxylipin patterns in obese, insulin‐resistant, sedentary women

**DOI:** 10.14814/phy2.14547

**Published:** 2020-09-01

**Authors:** Dmitry Grapov, Oliver Fiehn, Caitlin Campbell, Carol J. Chandler, Dustin J. Burnett, Elaine C. Souza, Gretchen A. Casazza, Nancy L. Keim, Gary R. Hunter, Jose R. Fernandez, W. Timothy Garvey, Charles L. Hoppel, Mary‐Ellen Harper, John W. Newman, Sean H. Adams

**Affiliations:** ^1^ CDS‐Creative Data Solutions Davis CA USA; ^2^ West Coast Metabolomics Center University of California Davis CA USA; ^3^ United States Department of Agriculture‐Agricultural Research Service Western Human Nutrition Research Center Davis CA USA; ^4^ Department of Kinesiology California State University Sacramento CA USA; ^5^ Department of Nutrition University of California Davis CA USA; ^6^ Department of Nutrition Sciences University of Alabama Birmingham AL USA; ^7^ Human Studies Department University of Alabama Birmingham AL USA; ^8^ Pharmacology Department Case Western Reserve University Cleveland OH USA; ^9^ Department of Biochemistry, Microbiology and Immunology, and Ottawa Institute of Systems Biology University of Ottawa Ottawa ON Canada; ^10^ Arkansas Children’s Nutrition Center Little Rock AR USA; ^11^ Department of Pediatrics University of Arkansas for Medical Sciences Little Rock AR USA

**Keywords:** oxylipid, lipoxygenase, soluble epoxide hydrolase, polyunsaturated fatty acid, PUFA

## Abstract

Very little is known about how metabolic health status, insulin resistance or metabolic challenges modulate the endocannabinoid (eCB) or polyunsaturated fatty acid (PUFA)‐derived oxylipin (OxL) lipid classes. To address these questions, plasma eCB and OxL concentrations were determined at rest, 10 and 20 min during an acute exercise bout (30 min total, ~45% of preintervention V̇O_2peak_, ~63 W), and following 20 min recovery in overnight‐fasted sedentary, obese, insulin‐resistant women under controlled diet conditions. We hypothesized that increased fitness and insulin sensitivity following a ~14‐week training and weight loss intervention would lead to significant changes in lipid signatures using an identical acute exercise protocol to preintervention. In the first 10 min of exercise, concentrations of a suite of OxL diols and hydroxyeicosatetraenoic acid (HETE) metabolites dropped significantly. There was no increase in 12,13‐DiHOME, previously reported to increase with exercise and proposed to activate muscle fatty acid uptake and tissue metabolism. Following weight loss intervention, exercise‐associated reductions were more pronounced for several linoleate and alpha‐linolenate metabolites including DiHOMEs, DiHODEs, KODEs, and EpODEs, and fasting concentrations of 9,10‐DiHODE, 12,13‐DiHODE, and 9,10‐DiHOME were reduced. These findings suggest that improved metabolic health modifies soluble epoxide hydrolase, cytochrome P450 epoxygenase (CYP), and lipoxygenase (LOX) systems. Acute exercise led to reductions for most eCB metabolites, with no evidence for concentration increases even at recovery. It is proposed that during submaximal aerobic exercise, nonoxidative fates of long‐chain saturated, monounsaturated, and PUFAs are attenuated in tissues that are important contributors to the blood OxL and eCB pools.

## Graphical abstract

The body's response to exercise involves shifts in hundreds of metabolites in tissues and blood. This study determined how specialized signaling lipids called oxylipins and endocannabinoids "respond" to an acute bout of exercise in women. Reductions in blood concentrations of many of these metabolites could signal that during submaximal aerobic exercise, flux through "non‐oxidative" pathways for fat metabolites is reduced.

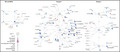

## 1 INTRODUCTION

Insulin resistance and type 2 diabetes mellitus (T2DM) are associated with alterations in lipid metabolism. For example, under these conditions there are increased indices of incomplete long‐chain fatty acid (LCFA) β‐oxidation in the blood (Adams et al., 2009; Mihalik et al., 2010), and incomplete β‐oxidation is more evident in cultured myotubes isolated from insulin‐resistant persons compared to insulin‐sensitive individuals (Aguer et al., 2015). Furthermore, insulin resistance in adipocytes can promote higher basal lipolysis (Abdul‐Ghani, Molina‐Carrion, Jani, Jenkinson, and Defronzo, 2008; Lofgren, Hoffstedt, Naslund, Wiren, & Arner, 2005). Taken together, these observations indicate that cellular pools of fatty acids including polyunsaturated fatty acids (PUFA) are higher with insulin resistance and T2DM, which could further feed nonoxidative pathways including biosynthesis of endocannabinoids (eCBs) and oxylipins (OxL).

Evidence supporting this idea comes from our previous report comparing plasma eCBs and OxL in weight‐matched, overnight‐fasted obese African‐American women with and without T2DM (Grapov, Adams, Pedersen, Garvey, & Newman, 2012). T2DM was characterized by: (a) higher fatty acid concentrations from all classes (saturated and unsaturated LCFAs, PUFAs, and very long‐chain fatty acids [VLCFAs]), (b) ~30% to 60% increased markers of stearoyl‐CoA desaturase activity, (c) ~70% enhanced indices of elongase of VLCFA‐2 (ELOVL2) activity, (d) ~30% to 60% lower markers of peroxisome‐associated Sprecher pathway chain shortening (SPCS), (e) ~44% to 127% higher circulating concentrations of epoxides derived from linoleic acid, alpha‐linolenic acid, and arachidonic acid (AA), (f) ~30% to 40% increases in select AA‐derived dihydroxyeicosatrienoates (DiHETEs), and (g) ~86% higher concentration of the fatty acid ketone 13‐keto‐9Z,11E‐octadecadienoic acid (13‐KODE), produced from dehydrogenation of 13‐hydroxy‐9Z,11E‐octadecadienoic acid (13‐HODE). In that study, T2DM was also characterized by higher plasma eCB‐like metabolites, including many N‐acylethanolamides (~45% to 100%) and N‐oleoylglycine (NO‐Gly) (148%). Strikingly, participant scores generated by orthogonal partial least squares discriminate analysis (OPLS‐DA)—using T2DM discriminating metabolites—were strongly correlated to overnight‐fasted blood glucose concentrations (*r* = .7, *p* <.0001). This illustrates a potential for connection between glucose homeostasis and metabolism of OxLs and eCBs.

Altered metabolism of OxLs and eCBs could, in theory, manifest even more when whole‐body fatty acid flux accelerates, as occurs during aerobic exercise. Moderate exercise increases lipolysis, and plasma LCFA concentrations drop significantly due to active tissue uptake from the blood pool (see, e.g., Grapov et al., 2019; Romijn et al., 1993). In moderately active healthy individuals, 20 min of cycling at 80% maximal workload decreased plasma AA and increased AA epoxide catabolic products, but did not change AA epoxide concentrations (Giordano, Newman, Pedersen, Ramos, & Stebbins CL et al., 2011). More recently, Nieman and Mitmesser (2017) described OxL patterns in response to a 75 km time trial in trained normal weight cyclists, and Stanford et al. (2018) reported OxL changes with submaximal aerobic exercise in healthy adults. Yet, no studies have addressed exercise OxL patterns by comparing metabolically unhealthy and healthy states, and there is a paucity of information regarding acute changes in eCBs with moderate exercise.

We recently described acute exercise‐ and recovery‐associated plasma patterns of acylcarnitines (Zhang et al., 2017) and “global” metabolite profiles (Grapov et al., 2019) in a cohort of insulin resistant, sedentary obese women, before and after a ~14‐week fitness and weight loss intervention that significantly improved insulin sensitivity (Campbell et al., 2014). The current paper complements and further characterizes the metabolic profile of the cohort, by analyzing comprehensive panels of OxLs and eCBs at 0, 10, and 20 min of a 30 min sub‐maximal exercise bout, and then following 20 min of recovery. Considering the observation that overnight‐fasted blood OxL and eCB patterns are responsive to metabolic health (Grapov et al., 2012), we reasoned that improved insulin sensitivity following a fitness and weight loss intervention would significantly alter (“normalize”) fasting OxL and eCB patterns, and lead to distinct exercise‐associated OxL and eCB metabolite patterns when compared to preintervention.

## 2 MATERIALS AND METHODS

### 2.1 Ethics Approval and Human Subjects Information

Extensive details regarding diet, recruitment, Test Week protocols, and other intervention‐associated aspects for this cohort are provided in our reports of oral glucose tolerance test (OGTT) metabolomics (Campbell et al., 2014), acute exercise acylcarnitine profiling (Zhang et al., 2017), and acute exercise impact on global metabolomics (Grapov et al., 2019). A succinct summary is provided here. All protocols were approved by the University of California at Davis Institutional Review Board, in alignment with the Declaration of Helsinki, and all subjects provided informed written consent. The study is listed in ClinicalTrials.gov (NCT01494025). Women who were 30–50 years of age, obese and insulin resistant were recruited. All participants were eumenorrheic, nonsmoking, and sedentary (typical planned exercise <30 min per week), with a body mass index (BMI) between 30 and 37.5 kg/m^2^. Insulin resistance at screening was defined as follows: (a) as per the American Diabetes Association guidelines for prediabetes, fasting glucose ≥100 and <126 mg/dL or 2‐hr OGTT glucose ≥140 and <199 mg/dL; and/or (b) a target Quantitative Insulin Sensitivity Check Index (QUICKI) score <0.315, Homeostasis Model Assessment (HOMA) >3.67, or logHOMA > 0.085. These measures were all highly correlated (Campbell et al., 2014). Exclusion criteria included clinical signs of infection, chronic disease, personal history of cardiovascular disease, elevated blood pressure (>130/85 mmHg), diabetes, regular medications other than oral contraceptives, and pregnancy or lactation. Sixteen participated through the first phase of the study; one subject was not adherent to the prescribed diet provided during either Test Week and was therefore excluded. Three subjects dropped following Test Week 1, prior to or during weight loss/exercise intervention, leaving 12 of 15 adherent subjects available for reexamination in Test Week 2.

### 2.2 Pre‐ and post‐intervention test week protocol

Participants completed testing before (“Test Week 1”) and after (“Test Week 2”) an exercise and weight loss intervention lasting 14–17 weeks (variable due to disparate menstrual cycle patterns: stages were matched pre‐ and post‐intervention) as previously discussed (Campbell et al., 2014; Zhang et al., 2017). During each Test Week, subjects refrained from exercise and were weight‐stable, with body mass determined daily and changes in provided calories made to maintain body mass within 5%.

#### 2.2.1 Test Week Diet

To minimize variability in metabolomics measures, during Test Week 1 and Test Week 2, participants were provided lot‐matched foods such that they ate identical diets for the Test Weeks (Campbell et al., 2014).

Peak Exercise Test (also described in Zhang et al., 2017 ): On Day 4 or 5 of each Test Week, a graded cycle ergometer test (SRM ergometer, Colorado Springs, CO) was performed to determine peak oxygen consumption (V̇O_2peak_). Participants arrived at the UC Davis Sports Medicine Clinic after consuming a standard breakfast (Menu 2 in: (Campbell et al., 2014)) 2–3 hr prior to exercise. During Test Week 1, participants received a resting ECG, a spirometry test, and a medical clearance exam to ensure there were no health issues precluding exercise. For the fitness test conducted in Test Week 1 and Test Week 2, participants completed a 5 min warm up, followed by a graded exercise test to exhaustion: initial workload of 50W, increased by 20W every 2 min until volitional fatigue. V̇O_2peak_ was determined as the highest V̇O_2_ (mL/kg/min) over a 30‐s period.

#### 2.2.2 Plasma metabolite profiling during submaximal exercise test

Forty‐eight hours after the peak exercise test, on Day 7 or 8 of the Test Weeks, participants reported following a 12‐hr overnight fast and having refrained from any moderate to vigorous physical activity since the V̇O_2peak_ test. Subjects were fitted with a HR monitor (Polar Vantage NV™ model #1901001, Polar Electro Inc.). An intravenous catheter was placed in an arm vein (typically antecubital). The first blood sample was taken 5 min after catheter placement. After a brief 5‐min warm‐up involving pedaling without workload, subjects were fitted with headgear and nose clip for metabolic cart measurements. During Test Week 1, the tension on the cycle ergometer (Monark 828E, Sweden) was set to elicit an individualized workload at 45% V̇O_2peak_ (average 64W) determined from the peak exercise test. Participants pedaled at the appropriate cadence of 50–60 rpm for 30 min. Between 0 and 5 min of exercise, workload was slightly adjusted to ensure the subject was working at an intensity of 45% V̇O_2peak_. The apparatus was removed until a second calorimetry measurement between 15 and 20 min. Blood was sampled every 5 min throughout the entire protocol, as was HR; however, only a subset of samples was available for analysis of OxLs and eCBs reported herein. At 30 min, the workload was reduced to zero watts for a 5 min “cool down” during which subjects continued to pedal at a slow pace. Participants then moved to a chair where they rested for 15 min (between 35 and 50 min of the protocol). They continued to have blood drawn every 5 min; however, only the 50‐min sample was available for OxL and eCB analyses. Blood was collected into EDTA Vacutainers (Becton‐Dickinson), placed in ice, centrifuged for plasma, and plasma stored at −80°C. During Test Week 2, the same regimen was applied, but with the workload on the cycle ergometer matching the individual’s Test Week 1 workload to ensure equal muscle work.

### 2.3 Weight loss and fitness regimen

Subjects were prescribed a self‐selected calorie‐restricted diet (~500–600 kcal/day reduction) based on the 2005 Dietary Guidelines for Americans and targeting a 10% body mass loss (Campbell et al., 2014). A questionnaire (Baecke, Burema, & Frijters, 1982) was administered to assess self‐reported physical activity level, with a score of 5 for the lowest activity and 15 for the highest activity related to work, sport/exercise, and nonsport leisure categories; a score of 7 was used to calculate maintenance calories. Participants recorded daily food intake in diaries and received weekly counseling from a registered dietitian. Subjects were provided with a daily nutritional supplement (Bayer One‐a‐Day for Women) to assure adequate intake of essential vitamins and minerals. Body mass was measured weekly. Participants engaged in a prescribed exercise regimen of at least 4 times per week for the duration of the intervention. Over the first four intervention weeks, participants exercised aerobically 4 days per week for 30 min each (treadmill or cycle ergometer) at an intensity of 60%–70% of their maximal HR. During intervention weeks 5–8, exercise sessions increased to 40 min per session, 4 days per week and during intervention week 9 onward the intensity increased to 75% of maximal HR.

### 2.4 Analysis of Fatty Acid Methyl Esters

Plasma free fatty acids were measured as methyl esters by GC‐MS using internal standard methodology as previously described (15). Briefly, 20 µl of plasma was enriched with extraction surrogates including 20:3n3, and extracted with isopropanol/cyclohexane/ammonium acetate, solvent removed, and residues reconstituted in 1:1 methanol/toluene. Samples were enriched with 15:1n5 and fatty acids methylated with trimethylsilyl‐diazomethane in hexane (Sigma‐Aldrich, St. Louis, MO), dried under vacuum and reconstituted in hexane containing 23:0 for analysis. FAMEs were separated on an HP6890 GC equipped with a 30 m × 0.25 id × 0.25 µm DB‐225ms column (Agilent Technologies) and detected with a 5973N MSD with electron impact ionization. Analytes were quantified with ChemStation vE.02.14 software (Agilent) using internal standard methodologies against a 5‐ to 7‐point calibration curve bracketing all reported concentrations.

### 2.5 Oxylipin and endocannabinoid analysis

Plasma nonesterified oxylipins (OxL) and endocannabinoid and endocannabinoid‐like compounds (eCB) were isolated by solid phase extraction and quantified by LC‐MS/MS using internal standard methodology as previously described (Grapov et al., 2012). Briefly, after enrichment of 250 µl plasma with a suite of deuterated extraction surrogates, OxLs and eCBs were extracted using solid phase extraction on Oasis HLB (10 mg, Waters) stationary phase. Extracted samples were reconstituted in 50 µl of methanol containing 1,000 nM of the internal standard 1‐cyclohexyl‐3‐ureido dodecanoic acid (CUDA; Sigma‐Aldrich). Residues were separated during independent injections on a 2.1 × 150, 0.7 µm Aquity BEH column (Waters) and detected using either negative mode (OxL) or positive mode (eCB) electrospray ionization and multi reaction monitoring on an API 4000QTRAP (Sciex).

### 2.6 Analytical Quality Controls

For both GC and LC analyses analytical quality assurance and quality control measures included the following: sample randomization to 11 processing batches; processing batch inclusion of reference plasma replicates and blanks; processing batches were randomized prior to GC‐MS analysis; the use of isotopically labeled or rare analytes as extraction surrogates and/or derivatization controls. All reported data were bracketed by calibration curves, had greater than 3:1 signal to noise, and replicate precision was generally less than 25% for analytes with intensities greater than 10× their detection limit.

### 2.7 Statistics, analysis and visualization

Analyses were conducted using the R programming language (v3.6.0). The previously developed analysis strategy (Grapov et al., 2019) was adapted to identify: (1) clusters of related lipid time course patterns (t0–t50 min); (2) exercise‐associated effects and changes in lipids: (a) between t0 and t10 min of exercise regardless of weight loss status, (b) comparing pre‐versus post‐intervention concentrations, and (c) comparing pre‐ versus post‐intervention at recovery (t50 min, exercise ceased 20 min prior). Biochemical network mapping was used to visualize the statistical analysis results.

### 2.8 Time course clustering

Related patterns in lipid time course profiles were identified using time series clustering with optimizations for the dynamic time warping distance (R package dwtclust; Sardá‐Espinosa, 2018). Acute exercise time course patterns (t0, t10, t20 and t50 min) were grouped into related patterns using partitional clustering. Individual lipid concentrations were expressed as ratios to their baselines (t0) and summarized as medians for pre‐ and post‐intervention samples. An optimal number of clusters was identified based on minimization of inter‐cluster distances for seven clusters, shape‐based distance (Paparrizos & Gravano, 2015), partitioning around medoids, centroids, and *z*‐score scaling.

### 2.9 Statistical Analysis

**Figure 1:**
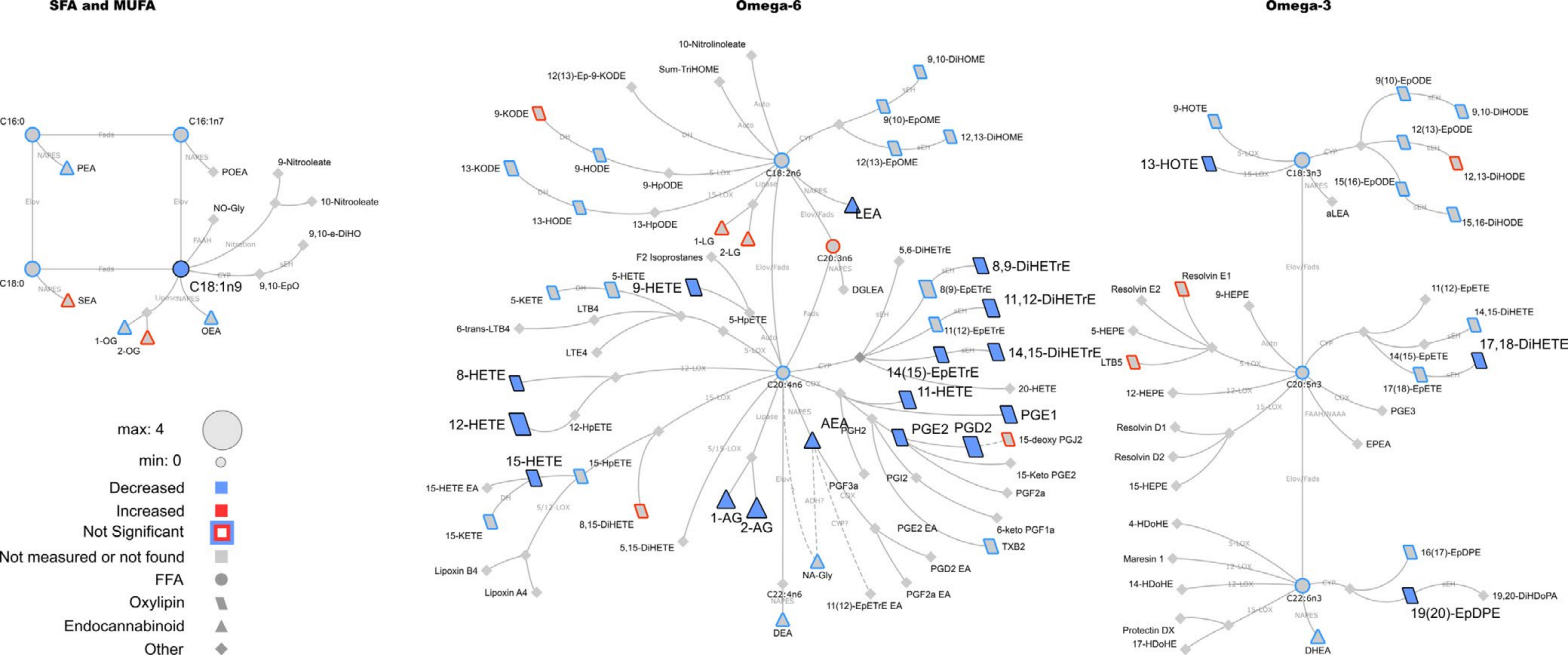
Lipid networks, illustrating acute exercise effects (0–10 min) on plasma metabolites in adult women, regardless of fitness and weight loss intervention. The biochemical network displays precursor–product relationships for fatty acids, oxylipins (OxL) and endocannabinoids (eCB) for related subclasses of fatty acid precursors including saturated and monounsaturated, omega‐6 and omega‐3 polyunsaturated fatty acids. The magnitude, direction and significance of changes are denoted by node size (the magnitude of the spearman correlation over the 10 min), border or fill colors (red, increased plasma concentration; blue, reduced plasma concentration), respectively. Significant changes are shown for lipids with *p*_FDR_ values ≤.05 from mixed effects models. Non‐measured or undetected species are displayed in gray symbols with no borders

**Figure 2:**
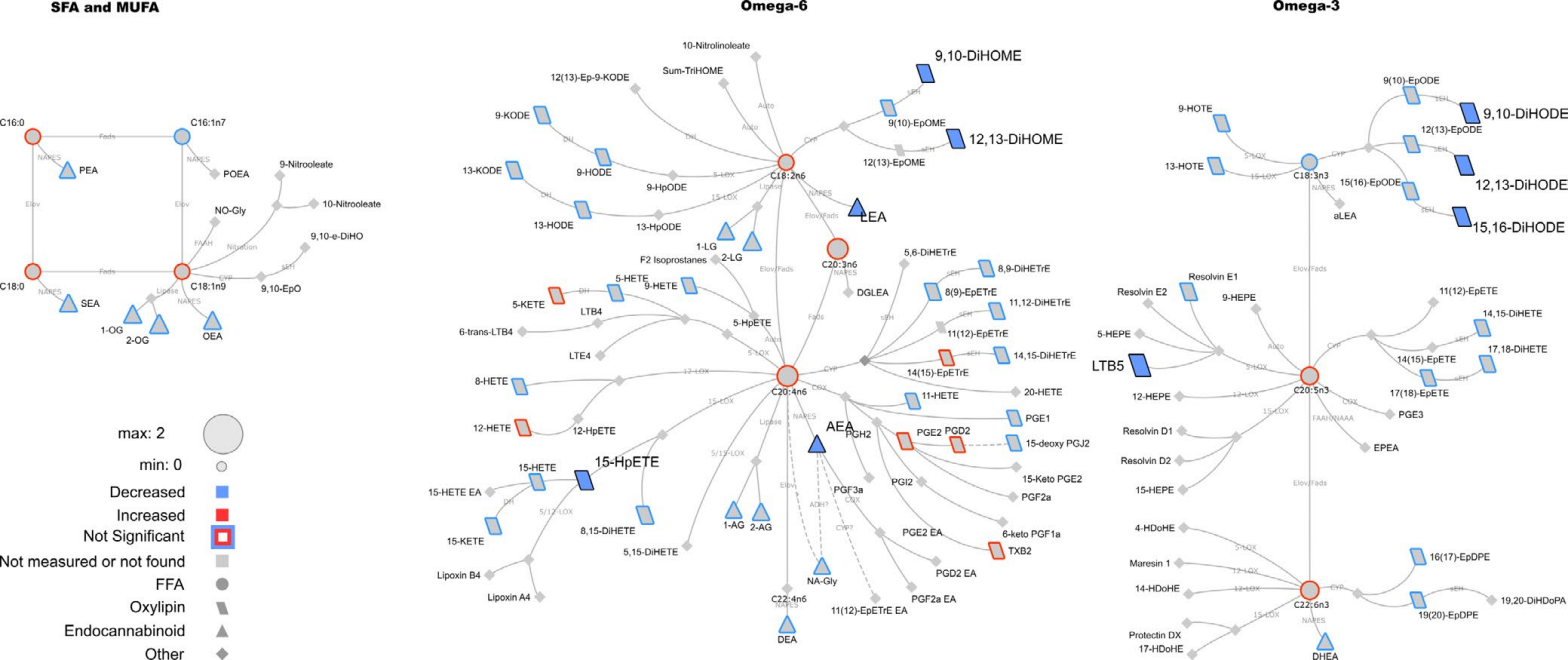
Lipid networks, illustrating the effect of fitness and weight loss intervention on postexercise recovery plasma metabolite patterns in adult women (at 20 min postexercise). The biochemical network displays precursor–product relationships for fatty acids, oxylipins (OxL) and endocannabinoids (eCB) for related subclasses of fatty acid precursors including saturated and monounsaturated, omega‐6 and omega‐3 polyunsaturated fatty acids. The magnitude, direction and significance of postintervention versus preintervention differences are denoted by node size, border or fill colors (red, higher concentration postintervention vs. preintervention; blue, lower concentration postintervention vs. preintervention), respectively. Significant changes are shown for lipids with *p* ≤.05 from Kruskal–Wallis nonparametric tests. Nonmeasured or undetected species are displayed in gray symbols with no borders

Exercise‐associated effects over the first 10 min of the bout were identified based on mixed effects models, with subjects as the random term. Residuals from a model for intervention effects, with subjects as the random term, were used to summarize intervention‐adjusted changes in lipid concentrations during exercise (see Table 1 and Figure 1). These patterns represent acute shifts in metabolites, regardless of intervention status. The magnitude and direction of the exercise effects were expressed as the fold‐change in means between t0 and t10. To compare which metabolites displayed intervention‐associated changes independent of acute exercise patterns, mixed effects models for t0 to t10 min were calculated, with subjects as the random term. Residuals from a model for time with subjects as the random term were used to summarize time‐adjusted differences in lipid concentrations between pre‐ and post‐intervention samples during exercise (see Figure 2). The magnitude and direction of the intervention effects were expressed as the fold‐change in means between pre‐ and post‐intervention measurements.

**Table 1:** Plasma metabolites with concentrations that changed significantly during a fixed workload exercise bout (0–10 min) in women, regardless of fitness and weight loss intervention

Name	Class	Type	*p*‐value	*p*_FDR_ –value	t0 (baseline, preexercise)	t10 (10 min into exercise bout)	t10 relative to t0
12(13)‐EpOME	OxL	C18‐Epoxides	.0367	.0981	6.216 ± 0.95	4.208 ± 0.7	0.68
13‐HOTE	OxL	C18‐Hydroxyl	.0087	.0346	1.142 ± 0.16	0.774 ± 0.1	0.68
13‐KODE	OxL	C18‐Ketone	.0156	.0507	6.604 ± 1.17	4.032 ± 0.67	0.61
LEA	eCB	C18‐NAE	1.37E‐05	.0002	2.747 ± 0.29	1.508 ± 0.2	0.55
Dihomo GLA EA	eCB	C18‐NAE	.0129	.0469	0.455 ± 0.06	0.306 ± 0.04	0.67
11,12‐DiHETrE	OxL	C20‐diols	1.37E‐09	1.25E‐07	0.254 ± 0.03	0.099 ± 0.01	0.39
14,15‐DiHETrE	OxL	C20‐diols	1.99E‐07	9.05E‐06	0.382 ± 0.05	0.162 ± 0.03	0.42
8,9‐DiHETrE	OxL	C20‐diols	2.61E‐06	5.93E‐05	0.114 ± 0.01	0.057 ± 0.01	0.50
17,18‐DiHETE	OxL	C20‐diols	8.93E‐06	.0001	1.252 ± 0.12	0.697 ± 0.09	0.56
19,20‐DiHDPA	OxL	C20‐diols	.0004	.0029	0.371 ± 0.05	0.203 ± 0.03	0.55
17(R)‐HDoHE	OxL	C20‐diols	.0322	.0887	1.093 ± 0.23	0.643 ± 0.15	0.59
19(20)‐EpDPE	OxL	C20‐epoxides	.0005	.0031	0.613 ± 0.08	0.324 ± 0.06	0.53
14(15)‐EpETrE	OxL	C20‐epoxides	.0015	.0085	0.344 ± 0.05	0.185 ± 0.03	0.54
8(9)‐EpETrE	OxL	C20‐epoxides	.0225	.0661	0.879 ± 0.33	0.325 ± 0.06	0.37
17(18)‐EpETE	OxL	C20‐epoxides	.0415	.1050	0.154 ± 0.03	0.101 ± 0.02	0.65
12‐HETE	OxL	C20‐Hydroxyl	9.23E‐06	.0001	39.106 ± 9.43	9.37 ± 2.23	0.24
12(S)‐HEPE	OxL	C20‐Hydroxyl	4.76E‐05	.0005	5.394 ± 1.38	1.371 ± 0.27	0.25
15(S)‐HETrE	OxL	C20‐Hydroxyl	.0002	.0015	0.375 ± 0.05	0.205 ± 0.03	0.55
15‐HETE	OxL	C20‐Hydroxyl	.0016	.0085	1.333 ± 0.24	0.658 ± 0.13	0.49
11‐HETE	OxL	C20‐Hydroxyl	.0024	.0123	0.392 ± 0.1	0.156 ± 0.04	0.40
9‐HETE	OxL	C20‐Hydroxyl	.0034	.0155	0.561 ± 0.13	0.253 ± 0.04	0.45
5(S)‐HEPE	OxL	C20‐Hydroxyl	.0069	.0286	0.364 ± 0.03	0.271 ± 0.02	0.74
8‐HETE	OxL	C20‐Hydroxyl	.0123	.0467	0.605 ± 0.11	0.371 ± 0.06	0.61
5‐HETE	OxL	C20‐Hydroxyl	.0197	.0597	1.315 ± 0.32	0.693 ± 0.12	0.53
15‐HpETE	OxL	C20‐Hydroxyl	.0235	.0669	0.678 ± 0.11	0.452 ± 0.07	0.67
2‐AG	eCB	C20‐MAG	.0033	.0155	20.627 ± 6.93	5.954 ± 1.24	0.29
1‐AG	eCB	C20‐MAG	.0136	.0477	2.589 ± 0.94	0.923 ± 0.16	0.36
AEA	eCB	C20‐NAE	1.17E‐06	3.54E‐05	0.996 ± 0.11	0.493 ± 0.07	0.49
DHEA	eCB	C22‐NAE	.0390	.1014	0.178 ± 0.02	0.124 ± 0.02	0.70
C18:1n9	FA	MUFA	.0049	.0212	291.960 ± 54.61	145.782 ± 44.48	0.50
C18:1n7	FA	MUFA	.0174	.0545	16.384 ± 3.53	8.542 ± 2.9	0.52
PGE1	OxL	Prostaglandins	8.72E‐05	.0009	0.043 ± 0.01	0.018 ± 0.003	0.40
PGE2	OxL	Prostaglandins	.0002	.0015	0.205 ± 0.04	0.094 ± 0.01	0.46
PGD2	OxL	Prostaglandins	.0002	.0015	0.121 ± 0.03	0.034 ± 0.01	0.28
PGF2a/ (isoprostanes)	OxL	Prostaglandins	.0003	.0021	0.099 ± 0.01	0.058 ± 0.01	0.58
TXB2	OxL	Thromboxane	.0154	.0507	3.702 ± 0.75	2.205 ± 0.22	0.60

Values are means ± SEM, for concentrations in nM.

Abbreviations: eCB, endocannabinoid; FA, fatty acid; OxL, oxylipin.

Preweight loss and fitness intervention, *n* = 15.

Postweight loss and fitness intervention, *n* = 12.

Exercise‐associated effects were identified based on a mixed effects model, adjusting for intervention. Model residuals were used to summarize intervention‐adjusted changes in lipid concentrations during exercise. Reported values include: mixed effect model *p*‐values, false discovery rate‐adjusted *p*‐values (*p*_FDR_‐value), mean ± standard SEM for pre‐ and post‐ intervention and fold‐change (FC) in postrelative to preintervention mean concentrations. Only lipids with *p* ≤ .05 are reported.

Changes in pre‐ versus post‐intervention lipid concentrations at baseline (t0) and 20 min post‐exercise (rest/recovery, t50) were identified based on Kruskal–Wallis nonparametric tests (Hollander & Wolfe, 1973). Paired statistical tests were not appropriate since not all subjects had matched pre‐ and post‐intervention samples. The magnitude and direction of changes in lipid concentrations at baseline and recovery were expressed as the fold‐change in means between pre‐ and post‐intervention measurements.

False discovery rate adjusted *p*‐values (pFDR) were calculated for all tests according to Benjamini and Hochberg (1995). Significant exercise‐associated effects and changes in pre‐ versus post‐intervention during exercise were defined based on *p*_FDR_ ≤.05. Analysis of intervention effects at preexercise baseline and recovery did not identify any changes at *p*_FDR_ ≤ .05, and their significance was instead determined based on the *p* ≤ .05. This treatment can lead to increased false positives but is useful to discuss the biological implication of the observed changes as a whole.

### 2.10 Biochemical network mapping

**Figure 3:**
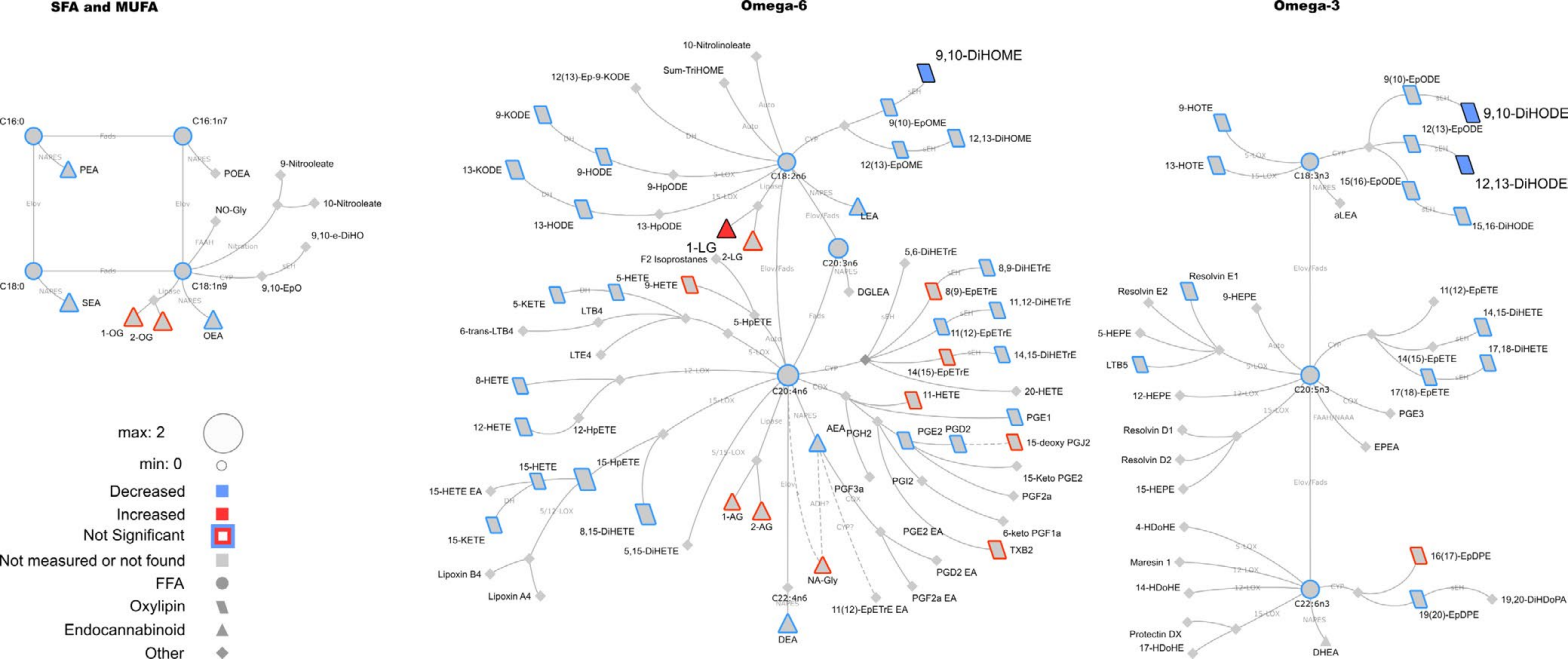
Lipid networks, illustrating the effect of fitness and weight loss intervention on plasma metabolite concentrations in overnight‐fasted adult women (preexercise bout time point). The biochemical network displays precursor‐product relationships for fatty acids, oxylipins (OxL) and endocannabinoids (eCB) for related subclasses of fatty acid precursors including saturated and monounsaturated, omega‐6 and omega‐3 polyunsaturated fatty acids. The magnitude, direction and significance of postintervention differences are denoted by node size, border or fill colors (red, higher concentration postintervention versus preintervention; blue, reduced concentration postintervention vs. preintervention), respectively. Significant changes are shown for lipids with *p* ≤ .05 from Kruskal–Wallis nonparametric tests. Nonmeasured or undetected species are displayed in gray symbols with no borders

**Figure 4:**
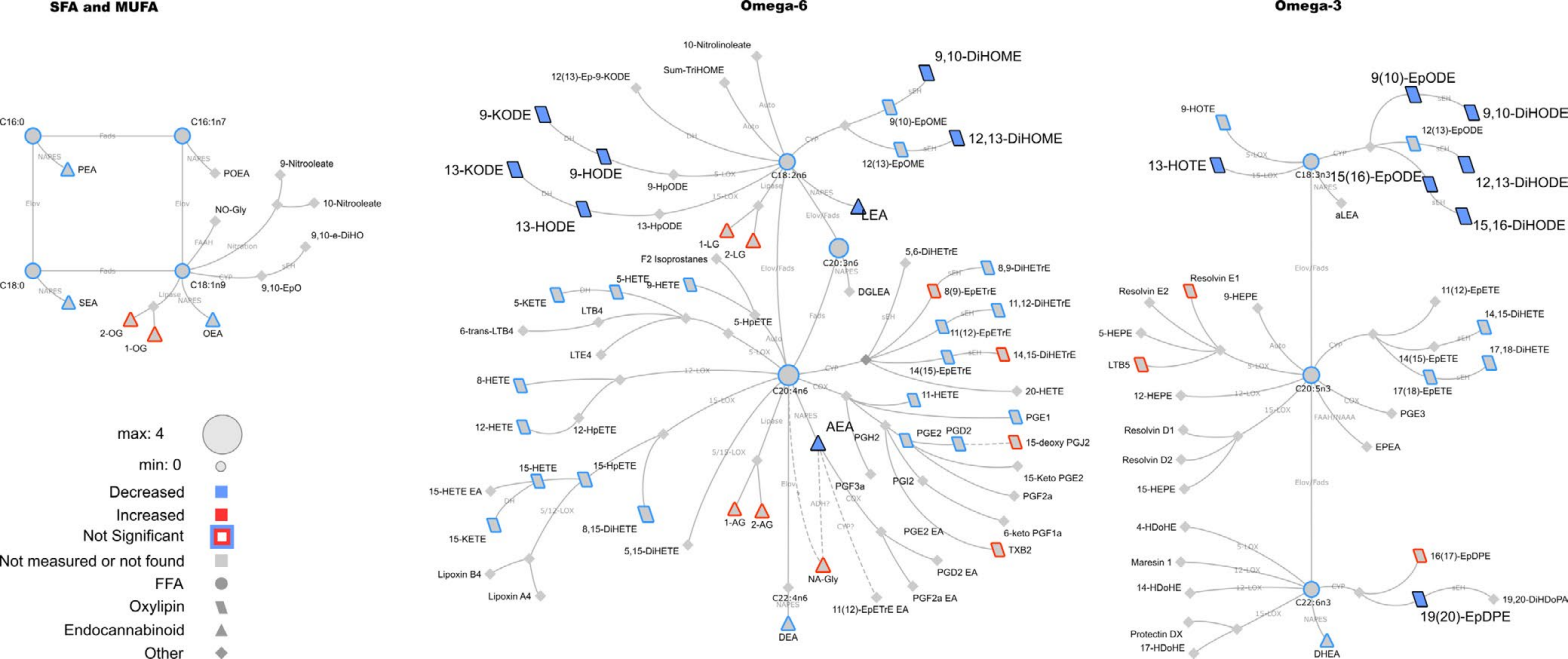
Lipid networks, illustrating effects of fitness and weight loss intervention plasma metabolite concentrations in adult women (using 0 and 10 min values together in the statistical model). The biochemical network displays precursor–product relationships for fatty acids, oxylipins (OxL) and endocannabinoids (eCB) for related subclasses of fatty acid precursors including saturated and monounsaturated, omega‐6 and omega‐3 polyunsaturated fatty acids. The direction and significance of postintervention versus preintervention differences are denoted by node size, border or fill colors (red, higher concentration postintervention vs. preintervention; blue, lower concentration postintervention vs. preintervention). Significant changes are shown for lipids with *p* ≤ .05 from mixed effects models. Nonmeasured or undetected species are displayed in gray symbols with no borders

Networks were calculated to summarize acute 10 min exercise‐associated changes regardless of intervention status (Figure 1), differences in pre‐ vs. post‐intervention metabolite concentrations at recovery (Figure 2), intervention effects using overnight‐fasted results (Figure 3) or using both t0 and t10 min data together in complementary *post hoc* analyses (Figure 4). Biochemical networks linking fatty acids, OxLs and eCBs based on precursor‐to‐product relationships were used to visualize the statistical analysis of each comparison. These networks are helpful to visualize the statistical significance and directionality of changes, while linking species based on their enzymatic relationships. This also highlights changes in downstream OxLs and eCBs from related subclasses of precursors: saturated fatty acids (SFA) and monounsaturated fatty acids (MUFA), omega‐6 and omega‐3 PUFA. Networks display the magnitude (node size), directionality of changes (node fill or border colors), and lipid class (node shape). Significant changes in lipids (see statistical analysis section for individual comparison thresholds) are denoted by node colors. Nodes for species failing to reach statistical significance are denoted with colored borders, and nonmeasured or undetected species are displayed in gray. All networks were visualized using Cytoscape (Shannon et al., 2003).

## 3 RESULTS

### 3.1 Body Mass loss and improvements in metabolic health indices following a fitness and dieting intervention

We previously reported that the intervention led to significant reductions in body weight (average ~6 kg, exclusively adipose), with increased fitness (V̇O_2peak_; from ~21 to ~26 ml kg^−1^ min^−1^) and ~48% improved insulin sensitivity (Campbell et al., 2014; Zhang et al., 2017). The following sections discuss: (a) patterns of acute exercise‐associated plasma metabolites (t0–t10 changes) regardless of pre‐ and post‐ weight loss and fitness intervention; (b) recovery metabolite profiles (at t50 [20 min postcessation of exercise]) comparing pre‐ and post‐intervention; (c) t0 baseline metabolites (preexercise, overnight‐fasted condition) comparing pre‐ and post‐intervention; (d) intervention‐associated changes in metabolite concentrations using statistical models including both t0 and t10 data. Results for all metabolites are presented in Materials S1 and S2; the latter may be found at: (https://data.nal.usda.gov/dataset/human‐blood‐plasma‐oxylipins‐endocannabinoids‐exercise‐patterns).

#### 3.1.1 Intervention‐independent, acute exercise‐associated plasma metabolite profiles

We first addressed how OxL and eCB metabolisms change with acute exercise in adult women, regardless of pre‐ versus post‐intervention status. A combination of statistical analysis and biochemical network mapping identified intervention‐independent time effects on OxL and eCB during 0–10 min of submaximal exercise (Figure 1). These patterns were modeled from data obtained both pre‐ and post‐ fitness and weight loss intervention, and hence represent the natural responses to submaximal exercise in women. As noted previously for “global” plasma metabolomics patterns in this cohort, many metabolites changed substantially at the 20‐min time point for reasons unknown, speculated to involve stress due to respiratory mask removal and a large blood draw (Grapov et al., 2019). Therefore, we have focused on the initial, acute phase of 10 min exercise for the current analysis. Patterns for all individual metabolites, broken out by pre‐ and post‐intervention, are provided in Materials S1. Significantly changed metabolites analyzed by univariate statistical analysis are noted in Table 1, and all metabolites are presented in Table S1.

1. *Oxylipins and fatty acids*. In general, blood OxL metabolites were decreased during the initial, acute phase of exercise when compared to preexercise baseline (Figure 1, blue symbols). Especially notable were the robust drops in prostaglandins (PGs) and HETE families of metabolites. These reductions were concurrent with significant decreases in precursor SFAs, MUFAs, and polyunsaturated fatty acids (PUFAs) including C18:2n6, C20:4n6, C18:3n3, C20:5n3, C22:6n3. There were some exceptions to the generally reduced OxL patterns. Concentrations of 9‐KODE (a keto octadecadienoate), 8,15‐DiHETE (a dihydroxy eicosatrienoate), 15‐deoxyPGJ2, LTB5 (a leukotriene), Resolvin E1, and 12,13‐DiHODE (a dihydroxy octadecadienoate) were modestly increased (red bordered metabolites), but the changes were not statistically significant and were highly variable (see, e.g., 12,13‐DiHODE pre‐ vs. post‐intervention, in Materials S1).
2. *Endocannabinoids*. Several eCBs were significantly reduced by acute exercise: e.g., 1‐AG and 2‐AG (arachidonoylglycerols), AEA (N‐arachidonoylethanolamine), LEA (linoleoylethanolamide) (Figure 1, blue symbols with black outline). OEA (oleoylethanolamide), DEA (docosatetraenylethanolamide), PEA (palmitoylethanolamide) and other eCB‐related patterns were less clearly reduced.

#### 3.1.2 Effect of weight loss and fitness intervention on plasma metabolites in the recovery state (at 20 min postcessation of a 30 min exercise bout)

We next sought to understand if the weight loss and fitness intervention altered the concentrations of metabolites at the recovery time point, 20 min after the exercise was stopped. A combination of statistical analysis and biochemical network mapping identified intervention‐dependent effects on OxLs and eCBs at 20 min of recovery following a 30 min exercise bout (Figure 2). Metabolites that differed significantly are presented in Table 2, and all metabolite results are in Table S2.

1. *Oxylipins and fatty acids*. Weight loss and fitness intervention was associated with reduced concentrations of DiHOME (dihydroxy octadecenoic acids) and DiHODE metabolites, as well as lower LTB5 and 15‐HpETE (a hydroxyeicosatrienoic acid), at recovery (Figure 2, blue symbols).
2. *Endocannabinoids*. Several eCBs were lower in concentration at the exercise recovery phase, following intervention: e.g., LEA and AEA differences were significantly different pre‐ versus post‐intervention (Figure 2, blue symbols with black outline), whereas the lower concentrations of OEA, 1‐OG and 2‐OG (oleoylglycerols), and 2‐LG (2‐linoleoylglycerol) at recovery in the postintervention state, were not statistically significant (Figure 2 and Materials S1).

**Table 2:** Plasma metabolites with concentration differences at the postexercise recovery time point, when comparing the pre‐ versus post‐ weight loss and fitness intervention periods in a cohort of women

Name	Class	Type	*p*‐value	*p*_FDR_‐value	Preintervention	Postintervention	Postconc. relative to Preconc.
9,10‐DiHODE	OxL	C18‐diols	.0007	.0624	0.313 ± 0.03	0.172 ± 0.02	0.55
15,16‐DiHODE	OxL	C18‐diols	.0022	.0704	9.413 ± 0.52	5.789 ± 0.72	0.61
12,13‐DiHODE	OxL	C18‐diols	.0024	.0704	0.208 ± 0.02	0.119 ± 0.01	0.57
12,13‐DiHOME	OxL	C18‐diols	.0031	.0704	7.579 ± 0.57	4.749 ± 0.52	0.63
9,10‐DiHOME	OxL	C18‐diols	.0135	.2461	27.519 ± 1.52	19.255 ± 2.42	0.70
LEA	eCB	c18‐NAE	.0327	.4260	6.266 ± 0.44	4.788 ± 0.46	0.76
15‐HpETE	OxL	c20‐Hydroxyl	.0448	.4423	1.042 ± 0.18	0.641 ± 0.21	0.61
AEA	eCB	C20‐NAE	.0328	.4260	2.152 ± 0.16	1.707 ± 0.13	0.79
LTB5	OxL	Leukotriene	.0421	.4423	0.244 ± 0.05	0.109 ± 0.02	0.45

Values are means ± SEM, for concentrations in nM measured at 20 min after cessation of the acute exercise bout. OxL, oxylipin; eCB, endocannabinoid.

Preweight loss and fitness intervention, *n* = 15.

Postweight loss and fitness intervention, *n* = 12.

Intervention‐associated effects were identified based on a Kruskal–Wallis nonparametric test. Reported values include: Kruskal–Wallis test *p*‐values, false discovery rate adjusted *p*‐values (*p*_FDR_‐value), mean ± SEM for pre and post intervention and fold‐change (FC) in post‐relative to preintervention mean concentrations. Only lipids with *p *≤ .05 are reported.

#### 3.1.3 Effect of weight loss and fitness intervention on plasma metabolites in the overnight‐fasted state

It was of interest to determine if weight loss and fitness intervention altered blood concentrations of OxL and eCB in the preexercise, baseline state. A combination of statistical analysis and biochemical network mapping identified intervention‐dependent differences in OxLs and eCBs following an overnight fast (Figure 3). Significantly changed metabolites are presented in Table 3, and all metabolite results provided in Table S3.

1. *Oxylipins and fatty acids*. Several OxLs were reduced in the postintervention state when compared to preintervention. For instance, 9,10‐diHOME, 9,10‐DiHODE, 12,13‐DiHODE were significantly lower (Figure 3, blue symbols with black outline). Concentrations of 9,10‐EpODE (an epoxyoctadecadienoic acid), 12,13‐DiHOME, 13‐KODE, 15,16‐DiHODE, 15,16‐EpODE, and 15 HpETE were also lower in the fasted state postintervention (see Materials S1), but these differences did not reach statistical significance in the t0 analysis.
2. *Endocannabinoids*. The fasting concentration of 1‐LG was higher postintervention (Figure 3, red symbol with black outline). Several other eCBs (e.g., 1‐OG, 2‐OG, and 2‐LG), were higher post‐intervention (Figure 3 and Material S1), but the differences were not statistically significant.

**Table 3:** Plasma metabolites with overnight‐fasted concentration differences when comparing the pre‐ versus post‐ weight loss and fitness intervention periods in a cohort of women

Name	Class	Type	*p*‐value	*p*_FDR_‐value	Preintervention	Postintervention	Postconc. relative to Preconc.
9,10‐DiHODE	OxL	C18‐diols	0.0218*	0.7521	0.326 ± 0.04	0.214 ± 0.04	0.65
12,13‐DiHODE	OxL	C18‐diols	0.0248*	0.7521	0.188 ± 0.01	0.137 ± 0.02	0.72
9,10‐DiHOME	OxL	C18‐diols	0.0480*	0.8139	23.447 ± 1.78	17.742 ± 2.11	0.76
1‐LG	eCB	C18‐MAG	0.0248	0.7521	6.345 ± 0.67	9.129 ± 0.81	1.45

Values are means ± SEM, for concentrations in nM.

Abbreviations: eCB, endocannabinoid; OxL, oxylipin.

Preweight loss and fitness intervention, *n* = 15.

Postweight loss and fitness intervention, *n* = 12.

Intervention‐associated effects were identified based on a Kruskal–Wallis nonparametric test. Reported values include: Kruskal‐Wallis test *p*‐values, false discovery rate adjusted *p*‐values (*p*_FDR_‐value), and fold‐change (FC) in post‐relative to pre‐intervention mean concentrations.

Only lipids with *p* ≤ .05 are reported.

* Significant intervention effect also observed in a mixed model test that included both t0 and t10 minute data, normalized to account for person‐to‐person differences in t0 absolute concentrations (see Figure 4).

#### 3.1.4 Effect of weight loss and fitness intervention on plasma metabolites: post hoc analysis using data from t0 and t10 min together

We recognized the high variability in concentrations of many metabolites in the overnight‐fasted state; to increase statistical sensitivity in terms of identifying intervention‐associated changes in metabolites, intervention effects were evaluated statistically using t0 and t10 min data together in a single model. This statistical analysis and biochemical network mapping identified intervention‐dependent effects on OxL and eCB concentrations (Figure 4), results complementary to those presented for the overnight‐fasted state, above. As an example, if a metabolite’s concentration was reduced significantly postintervention (lower at t0 and t10), this metabolite was colored with a blue symbol with black border in Figure 4 (see Materials S1 for t0–t10 profiles). Note that we looked for metabolites that displayed acute exercise bout patterns that were changed with intervention, based on a mixed effects models for t0–t10 including the interaction term between time and intervention. The short‐lived monohydroperoxy OxL, 15‐HpETE, was the only lipid which displayed a differential change during the first 10 min of exercise pre compared postintervention (interaction between exercise and intervention *p* = .029 and pFDR = .958).

1. *Oxylipins and fatty acids*. Concentrations of a myriad of OxL metabolites were lower post‐ versus pre‐intervention (see blue symbols, Figure 4): e.g., KODE and HODE family members, DiHOME metabolites, DiHODEs, 15,16‐EpODE, 13‐HOTE, and 19,20‐EpDPE. Looking at Materials S1, it can be seen that these metabolite classes track similarly with exercise, pre‐ versus post‐intervention, but at lower concentrations in the postintervention condition.
2. *Endocannabinoids*. The eCB concentrations were modestly impacted by weight loss and fitness intervention. For instance, AEA and LEA concentrations were lower in the postintervention state (Materials S1; Figure 4 blue symbols with black outline).

#### 3.1.5 Coordinated patterns of metabolites across the fasting‐to‐exercise‐to‐recovery spectrum

To deduce if specific pathways or metabolites are coregulated, one can leverage manual/visual inspection of individual metabolites across all time points in which samples were analyzed (see Materials S1), to identify common patterns. This may also be applied using visual inspection of Figures 1, 2, 3, 4 to see distinct precursor‐product network patterns under different states. An alternative, unbiased means to identify metabolites for which the plasma concentration profiles are similar is cluster analysis, using t0‐adjusted concentrations (pre‐exercise, fasted state), plus 10 min exercise, 20 min exercise, and 50 min (20 min postexercise, recovery state). Using this approach, seven clusters were generated using pre‐ and post‐intervention patterns independently (see Tables S4 and S5). Based on this approach, several interesting observations can be made:

1. Six of the seven cluster patterns include an exercise‐associated drop in metabolite concentrations at 10 min, highlighting the rapid response of whole‐body metabolism to acute exercise. This may signal rapid initial net tissue uptake of lipids and/or reduced production with onset of exertion.
2. Metabolites in Clusters 2 and 4 displayed a concentration “overshoot” during recovery, when compared to preexercise t0. This is in contrast to Clusters 1 and 5, in which recovery stage concentrations of metabolites had generally returned to t0 preexercise concentrations.
3. Metabolites in Clusters 3 and 7 tended to end in the recovery state at lower concentrations when compared to preexercise t0 concentrations, but patterns were complicated and in the case of Cluster 3, highly variable. High variability in individual metabolite responses were also seen in Cluster 6.
4. Some metabolites “switched” clusters when comparing the pre‐ vs. post‐intervention states, suggesting differential regulation coincident with changes in metabolic health (see Tables S4 and S5).

## 4 DISCUSSION

Exercise blood metabolomics analyses help unveil the metabolic landscape of physical activity, with the caveat that interpretations must be considered in light of exertion level, type and length of exercise, and time frame of measurements (e.g., see Brugnara et al., 2012; Chorell, Moritz, Branth, Antti, & Svensson, 2009; Lewis et al., 2010; Davison et al., 2018; Hansen et al., 2015; Mueller‐Hennessen et al., 2017; Nieman et al., 2014, 2015; Nieman, Sha, & Pappan, 2017; Peake et al., 2014; Zafeiridis et al., 2016). Our recent papers (Grapov et al., 2019; Zhang et al., 2017) and the results herein represent some of the first data that relate the exercise metabolome with improved metabolic health and fitness in the same cohort, and provide first‐ever information related to exercise metabolomics in women. In these studies, within the same cohort of women metabolite patterns were compared between a less healthy state (obesity, insulin resistance, and sedentary lifestyle) and a more healthy state characterized by increased insulin sensitivity and cardiorespiratory fitness following a weight loss and training intervention (described in: Campbell et al., 2014).

Previously in this cohort, we demonstrated that an acute submaximal exercise bout leads to rapid and robust changes in blood concentrations of acylcarnitines and small molecules reflective of intermediary metabolism (Grapov et al., 2019; Zhang et al., 2017). The current experiment extends those results through to OxL and eCB lipid mediators. Very few studies have considered OxL patterns during exercise (e.g., Giordano et al., 2011; Gollasch, Dogan, Rothe, Gollasch, & Luft, 2019; Nieman et al., 2019; Stanford et al., 2018) and no studies, to our knowledge, have determined how improved metabolic health and fitness in previously sedentary overweight individuals influences OxL or eCB patterns. Our results point to several important take‐home messages.

First, consistent with our previous results for acylcarnitines and small molecule patterns (Grapov et al., 2019; Zhang et al., 2017), exercise led to a rapid and significant change in concentrations of many blood OxL and eCB metabolites over the first 10 min of exertion, regardless of pre‐ or post‐weight loss and fitness intervention status. This speaks to the quick, adaptive shifts in metabolism that must take place to meet the energy needs associated with initiation of aerobic muscle work (e.g., ramp‐up of fat oxidation blended with glucose oxidation). Based on plasma metabolomics patterns, we have previously proposed that acute aerobic exercise leads to reduction of nonoxidative metabolic pathways for glucose (Grapov et al., 2019) and possibly reduces branched chain amino acid mitochondrial oxidation, at least transiently (Zhang et al., 2017). Since the current study design did not allow for flux determinations, the main tissue drivers of OxL and eCB blood profiles during acute exercise (0–10 min) or with recovery remain to be established.

Second, regardless of the pre‐ or post‐intervention state, exercise generally led to reductions in OxL metabolites, most notably members of the HETE, DiHETrE, and PG families as well as 13‐HOTE, 17,18‐DiHETE, and 19,20‐EpDPE. A survey of all metabolite patterns individually (Materials S1) further highlights acute reductions in most OxLs, remaining low or rising with time to concentrations about the same as the pre‐exercise sample (even if not statistically significant due to high variability). This contrasts with at least one previous report using a 75 km time trial model in male and female cyclists, in which OxL concentrations were generally increased immediately following the exercise challenge (Nieman et al., 2019). Qualitatively similar to our results, following a 40 min workout test at 70% of heart rate reserve in healthy men and women, Stanford et al. found that 13 blood OxLs were significantly reduced: 9‐oxoODE, 13‐oxoODE, 9‐HOTrE, 13‐HOTrE/13‐HOTrE(r), 13‐HODE, 9(10)‐EpOME, 9‐HEPE (hydroxyeicosa(penta)enoic acid), 12‐oxoETE, 14(15)‐EET, 12‐oxo‐LTB4, 8‐HDHA, 19(20)‐EpDPE and LTC4 (Stanford et al., 2018). In a different study, following 40 min cycling at 60% of maximal exercise intensity in 14 moderately active healthy individuals, there were no clear changes in OxL concentrations except for modest increases in 14(15)‐EET and14(15)‐DHET (Giordano et al., 2011). Taken together, the results from the literature and the current results indicate that exercise‐associated OxL blood patterns are likely dependent on exercise intensity or other related factors (e.g., sympathetic activation, stress or cytokine release), yielding variable results depending on study cohort phenotypes and experimental design.

The initial reduction in OxL concentrations in response to exercise in our cohort does not reflect limited availability of substrate fatty acids from the blood pool. Indeed, during acute exercise the plasma glycerol concentration increased (Grapov et al., 2019) and fatty acid concentrations dropped during exercise and rebounded in the recovery phase in this cohort (Grapov et al., 2019); and see Materials S1 for OxL‐related PUFA precursor patterns. Such a pattern is consistent with exercise‐associated lipolysis driven by sympathetic nervous system activation, coupled to accelerated muscle (and heart) LCFA uptake and combustion (Brooks, 1997; Hansen et al., 2015; Romijn et al., 1993). Another possibility is that enzymatic synthesis of OxL metabolites is reduced with acute aerobic exercise. One hypothesis is that plasma OxL reductions in the face of accelerated fatty acid flux with exercise is due in part to greater flow of precursor fatty acids toward oxidation in lieu of nonoxidative fates. The DiHETrEs, DiHETEs and DiHOMEs are produced through soluble epoxide hydrolase (sEH)‐dependent metabolism of antiinflammatory factors, like the cytochrome p450 (CYP) product 19,20‐EpDPE synthesized in the vascular endothelium and multiple peripheral tissues (Newman, Morisseau, & Hammock, 2005; Spector, 2009). On the other hand, HETEs, HODEs, LTB, LXA and HpODEs are products of lipoxygenases, cyclooxygenases, and/or the actions of reactive oxygen species (Mallat et al., 1999; Powell and Rokach, 1851). Gollasch et al. (2019), analyzing the plasma of a healthy nonobese group of five men and one woman undergoing treadmill exercise (2.7 km/h, 5% grade to exhaustion), did not observe differences in product/precursor ratios for metabolites reflective of sEH or CYP pathways in samples collected coincident with a heart rate of 150 beats per minute. In a *post hoc* evaluation based on product:precursor ratios for several epoxide/diols pairs (Table S6), there was a trend toward exercise‐associated increases in apparent sEH activity over the t0–t20 min time frame, followed by reduction at the t50 min recovery time point. Thus, there is little evidence that down‐regulation of OxL enzyme systems explains reduced blood OxLs with acute submaximal aerobic exercise in humans. The recovery‐associated dip in sEH surrogate indices appeared to be greater post‐intervention (Table S6), suggestive of a regulation of sEH biochemistry by metabolic health status.

The third key observation from the current study relates to 12,13‐DiHOME, an OxL previously reported as increased following a submaximal exercise bout (40 min workout at 70% of heart rate reserve or a 75 km time trial model in adults) (Nieman et al., 2019; Stanford et al., 2018) and mice (Stanford et al., 2018). This metabolite has been implicated in promotion of muscle fatty acid uptake, C2C12 myotube and whole‐body fatty acid oxidation, and increased myotube O_2_ consumption capacity in C2C12 myotubes (Stanford et al., 2018). Moreover, 12,13‐DiHOME has been shown to reduce myotube glucose uptake and inhibit insulin‐dependent Akt phosphorylation, and increase Ccl2 (i.e. MCP1) secretion (Huang et al., 2018). Brown adipose tissue is an important producer of this metabolite in mice, and administration of 12,13‐DiHOME promotes fatty acid uptake in brown fat (Lynes et al., 2017). Since in our exercise model there was no clear rise in plasma 12,13‐DiHOME, and there was no apparent change in the exercise study (Giordano et al., 2011) of R.M. Giordano et al. (JWN, personal communication), a systemic increase in this OxL is not a *sine qua non* for tissue fat uptake and combustion during aerobic exercise in humans. Based on results to date, the physiological role and regulation of this OxL remains equivocal. That said, weight loss and fitness intervention lowered fasting‐to‐exercise‐to‐recovery concentrations of 12,13‐DiHOME in the current study, which points to some type of connection between metabolic health status and 12,13‐DiHOME biochemistry.

Fourth, we previously described several OxL metabolite species to differ in overnight‐fasted T2DM women compared to nondiabetic women (Grapov et al., 2012); also see Section 1), so it is of interest to consider if those metabolites were also altered in the current cohort as their metabolic health improved with intervention. Preintervention, this study cohort was insulin resistant, sedentary and overweight; thus, metabolites increased in T2DM might be reduced by weight loss and fitness intervention. For instance, epoxides were higher by ~44% to 127% in T2DM (Grapov et al., 2012). However, no clear, significant differences were observed following intervention in the current cohort. T2DM was marked by ~30% to 40% increases in some diols, and herein we did observe lowered concentrations of several diols following intervention. Most convincingly, concentrations of 9,10‐DiHODE, 12,13‐DiHODE, and 9,10‐DiHOME were lower postintervention and displayed a statistically significant intervention effect. One hypothesis is that plasma diols reflect changes in sEH activities that associate with shifts in insulin sensitivity and metabolic health (see Xu et al., 2016). While a complete understanding of the role that sEH plays in glucose homeostasis has yet to emerge, sEH ablation increases pancreatic islet size and insulin signaling in high fat fed mice (Luria et al., 2011). Conversely, overexpression of this enzyme is associated with diabetes‐associated comorbidities including diabetic retinopathy and impaired wound healing (Hu et al., 2017; Sun et al., 2018). Despite the possibility that changes in sEH activity play a role in pre‐ versus post‐intervention OxL differences in our cohort, the sEH product/precursor ratios (diols:epoxides) for 9,10‐DiHODE, 12,13‐DiHODE, and 9,10‐DiHOME were not convincingly altered (~4%, 12%, 11% reductions, respectively). Another plasma metabolite, 13‐KODE, was higher by ~86% in T2DM women (Grapov et al., 2012); intervention in the current cohort led to a reduction in 13‐KODE at the overnight‐fasted and 10‐min exercise time points, but this was not statistically significant. Notably, the precursor metabolite 13‐HODE was also reduced at these time points postintervention. From these results, one can speculate that improved metabolic health associates with lower flux through (at least some) LOX pathways. We conclude that plasma concentrations of several OxL classes in the overnight‐fasted state appear to track metabolic health status in women, perhaps reflective of differences in substrates and sEH and LOX enzyme activities that are regulated by factors such as insulin action or lipid availability.

Finally, in addition to OxL metabolites, ω3 and ω6 fatty acids (as well as C16:0, C18:0, and C16/C18 MUFAs) can give rise to a variety of eCBs (Fezza et al., 2014; Tsuboi, Uyama, Okamoto Y, & Ueda, 2018). A number of studies have evaluated the impact of acute aerobic exercise on circulating eCBs in healthy adults, but no clear patterns have emerged. Further, analyses typically have not covered the entire spectrum of eCBs and derivatives. Sparling, Giuffrida, Piomelli, Rosskopf, and Dietrich (2003) reported ~50% increased AEA but no change in 2‐AG following 50 min of treadmill or cycling at 70%–80% of maximum heart rate in young men. In a study of male well‐trained cyclists, at 60 min of exercise at 55% of maximum power output on an ergometric bicycle, 2‐AG and AEA were unchanged, but there was a very modest increase in PEA and higher OEA (Heyman et al., 2012). In the same study, the moderate exercise was immediately followed by an intense time trial paradigm (work equal to 30 min at 75% of maximum power output), which did not further raise PEA but increased OEA and caused a small rise in AEA. Raichlen, Foster, Seillier, Giuffrida, and Gerdeman (2013) evaluated the impact of 30 min at varying work intensities (~45%–92% of maximum heart rate) in regular runners (men and women), and reported no change in 2‐AG but a significant increase in AEA at ~72% of maximum heart rate, other intensities displayed no change or equivocal results. In our study, there were reductions in ω6 fatty acid‐derived eCBs during the acute exercise phase, including LEA, 1‐AG, 2‐AG and AEA. Even when considering the 20 min and recovery patterns (Materials S1), there is little‐to‐no evidence for an exercise‐induced increase in plasma eCBs and derivatives. From this, and a review of the literature, we conclude that modest aerobic exercise in adults does not, by definition, trigger eCB increases in blood. It is possible that exercise intensity, training status, diet and energy balance, body composition/adiposity, time of collection, or exercise type impact select eCBs. More studies are needed that systemically address fitness level, exercise type, and temporal patterns during stepped workloads to evaluate fully the role of acute exercise on eCB biochemistry and physiology.

In conclusion, we observed that acute (0–10 min) submaximal exercise in women leads to generalized reductions in plasma concentrations of OxL and eCBs, which tend to recover to baseline fasting concentrations later in exercise and recovery but are not increased overall by exercise. There was no increase in 12,13‐DiHOME, a bioactive lipid previously hypothesized to be involved in exercise‐associated muscle fatty acid uptake and energetics. It is acknowledged that measurement of these lipid mediators in whole blood plasma may not report on localized or tissue‐specific regulation (i.e., in red blood cells, peripheral blood mononuclear cells, vasculature or microvasculature, adipose or liver), and the latter may be very important in OxL or eCB actions on inflammation responses, vascular tone, or other outcomes. Furthermore, the potential for lipoproteins to ferry and sequester complex lipids cannot be ignored, and is not addressed by measuring the total plasma pool. Future experiments can address these factors during exercise or with changes in metabolic health. Altogether, the results herein and those from the literature highlight that acute exercise drives significant changes in circulating pools of OxLs and eCBs, raising the possibility that that some of the acute and long‐term effects of exercise on whole‐body physiology involve local or systemic actions of these classes of complex lipids.

## CONFLICT OF INTEREST

The authors declare that they have no competing interests. S.H. Adams has previously served as a consultant to Abitec, LLC.

## AUTHORS’ CONTRIBUTIONS

SHA, CLH, GAC, NLK, JWN, GRH, JRF, WTG, MEH, OF, CC, CJC, and ECS: Exercise study design; SHA, CC, CJC, DJB, ECS, GAC, DG, and JWN: Conducted research; DG, SHA, JWN: Analyzed data; DG, JWN, SHA, Wrote manuscript. All authors edited and provided input to the manuscript.

## Supporting information



Material S1

Material S2
