# Supplementary figures and images for: Impact of a weight loss and fitness intervention on exercise‐associated plasma oxylipin patterns in obese, insulin‐resistant, sedentary women
Source: Physiol Rep. 2020 Sep 1;8(17):e14547. doi: 10.14814/phy2.14547 (PMC7460071; doi:10.14814/phy2.14547)

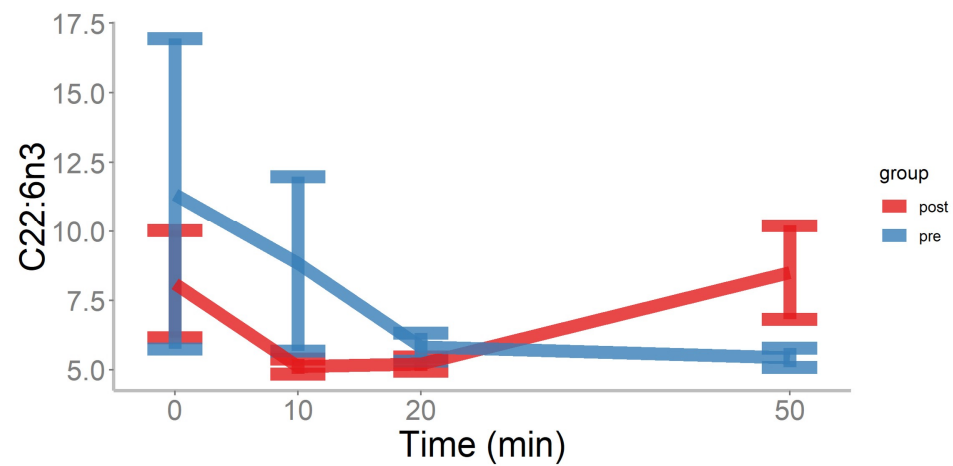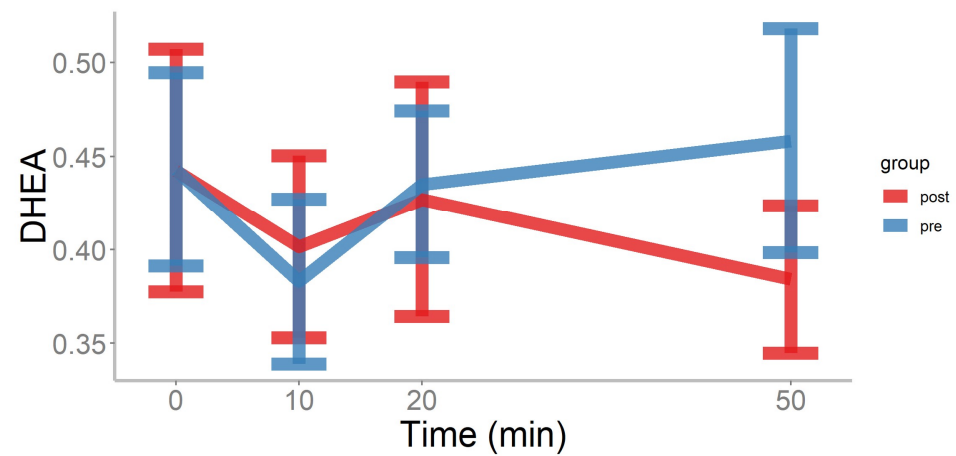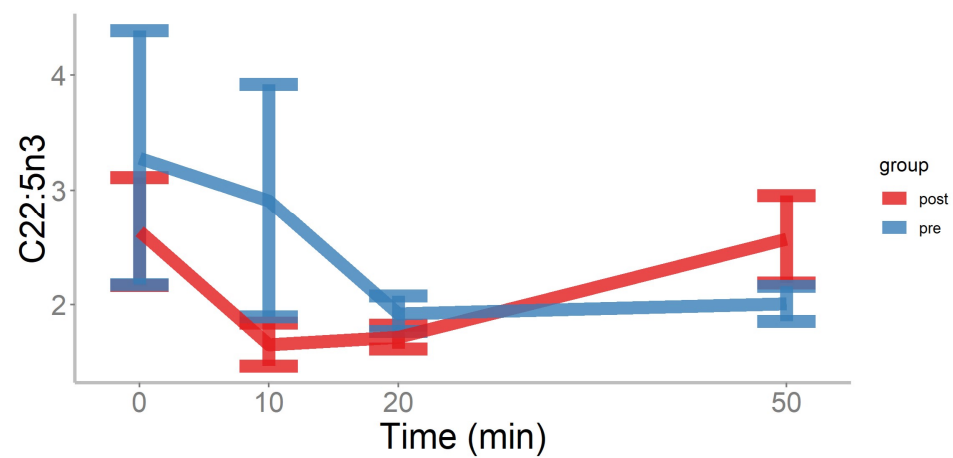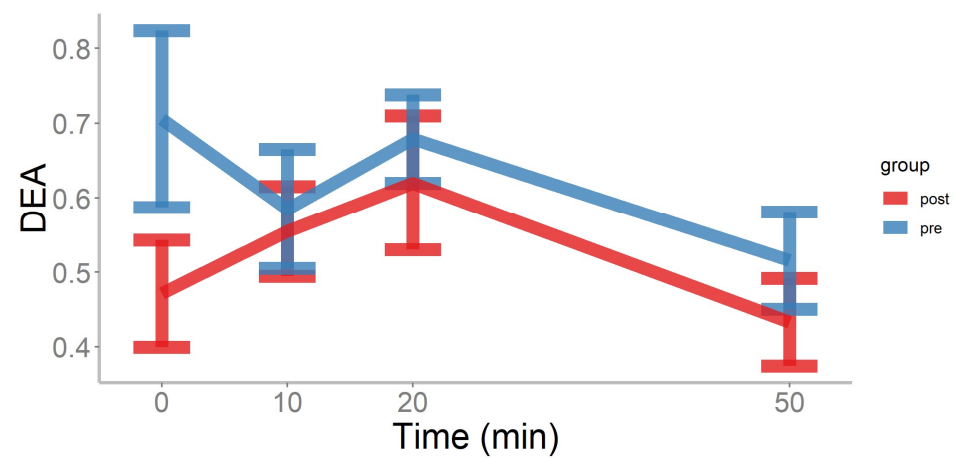

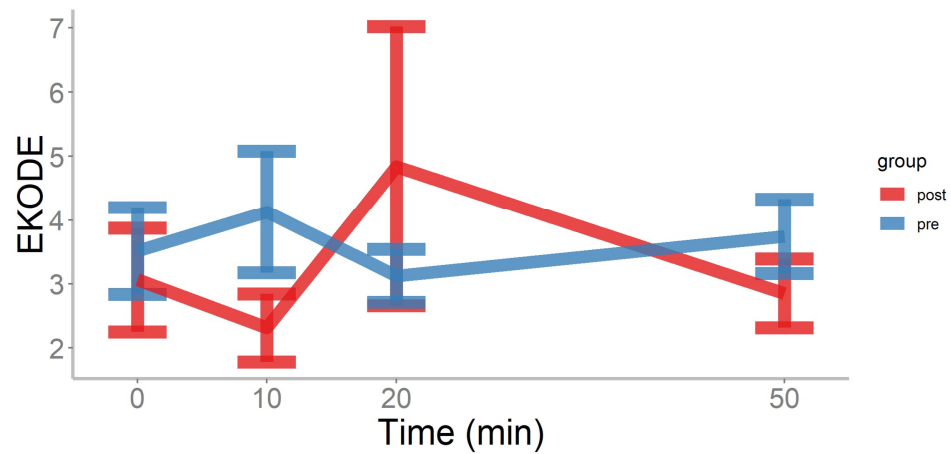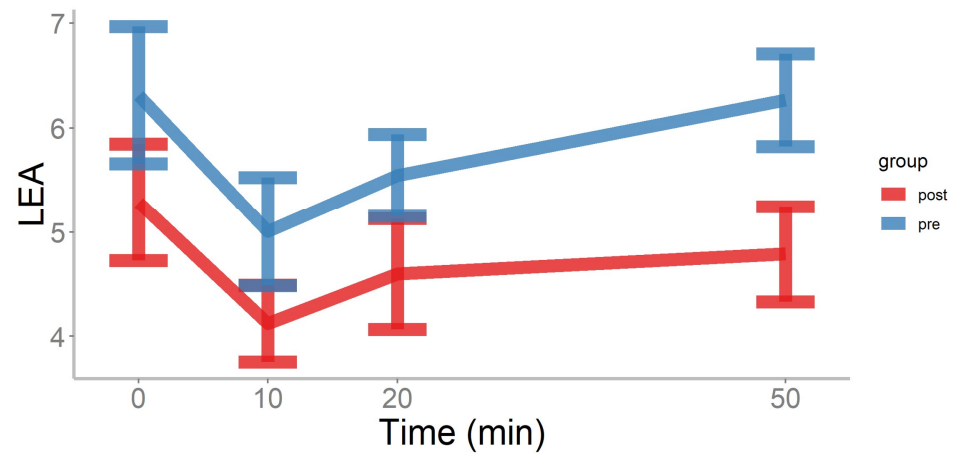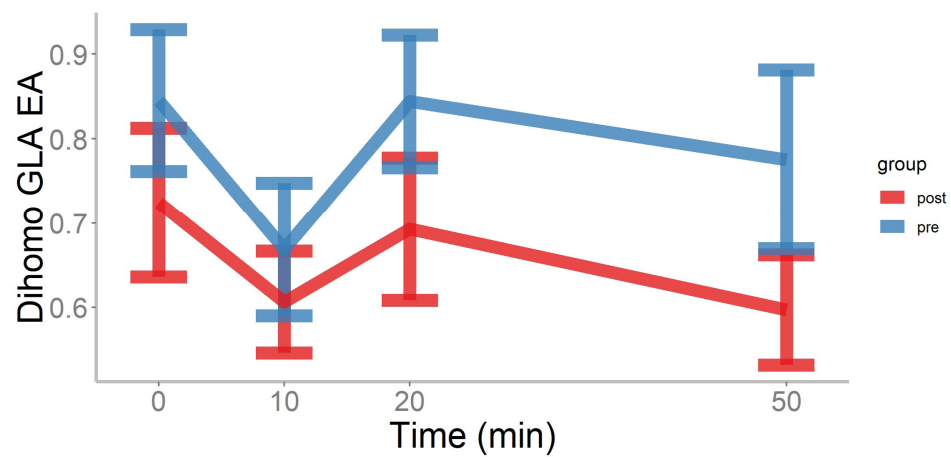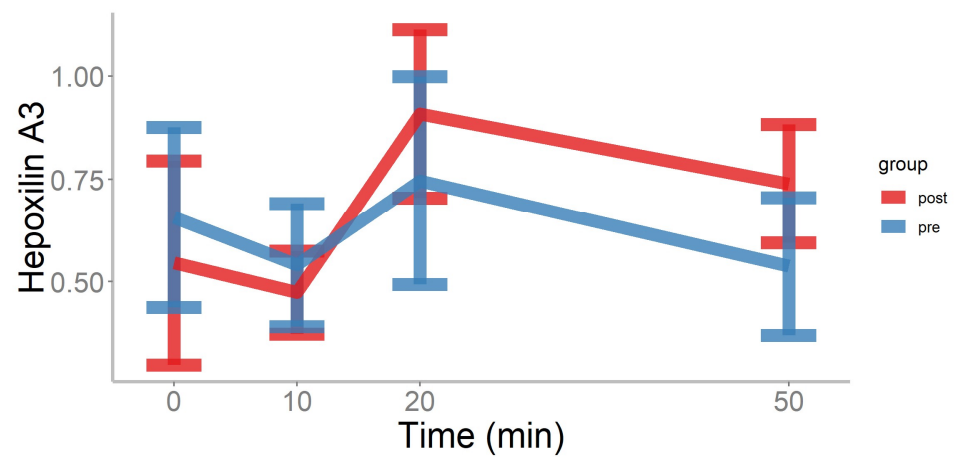

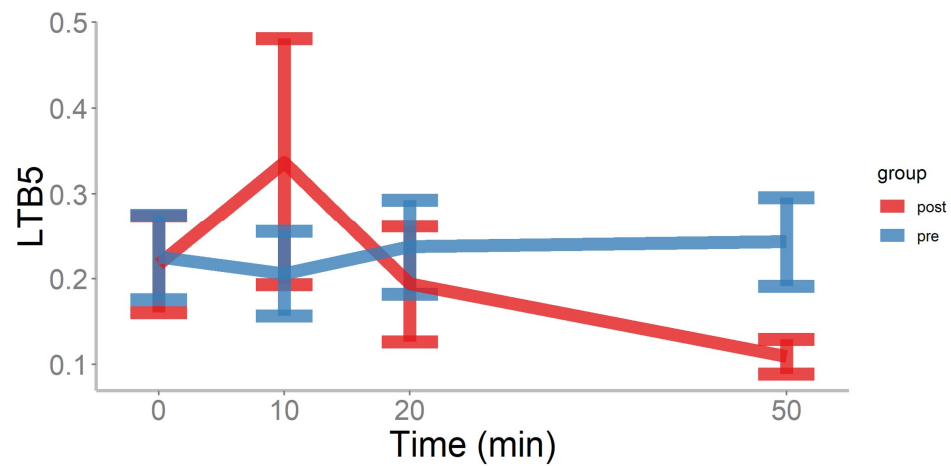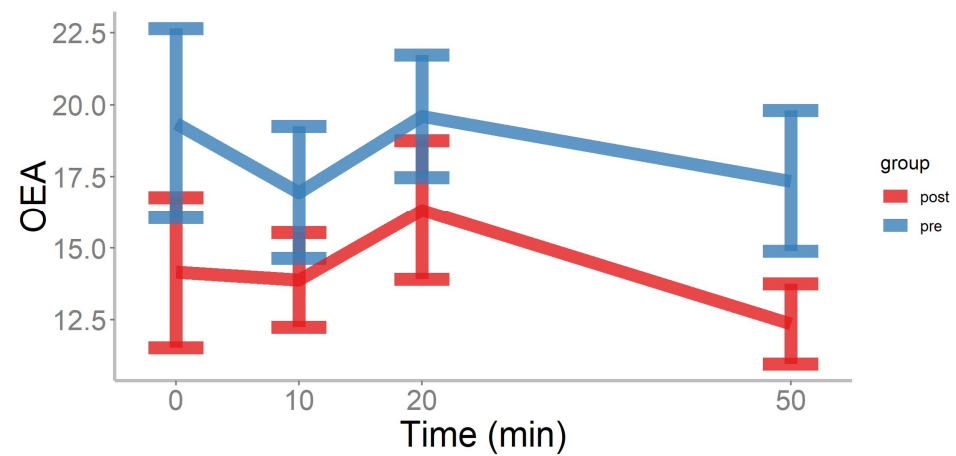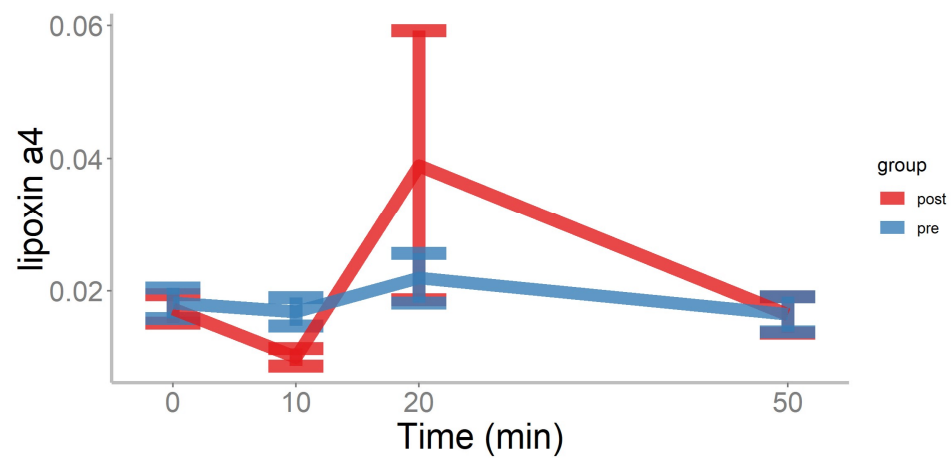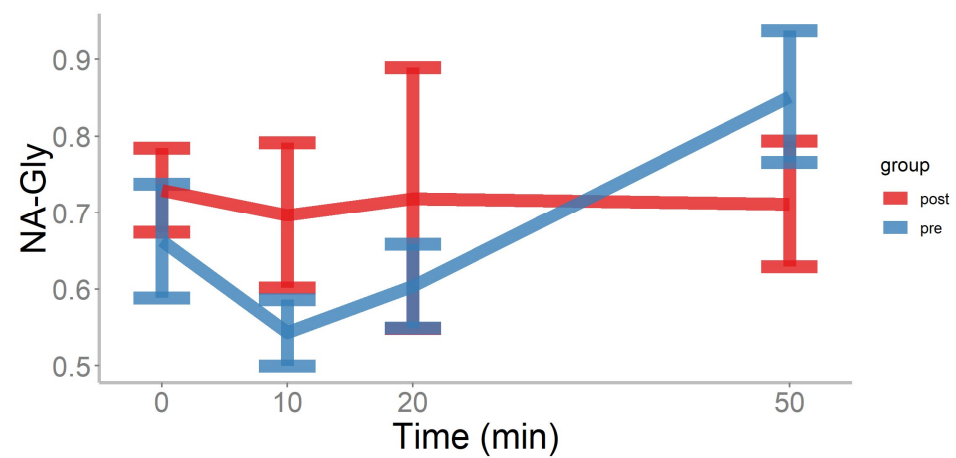

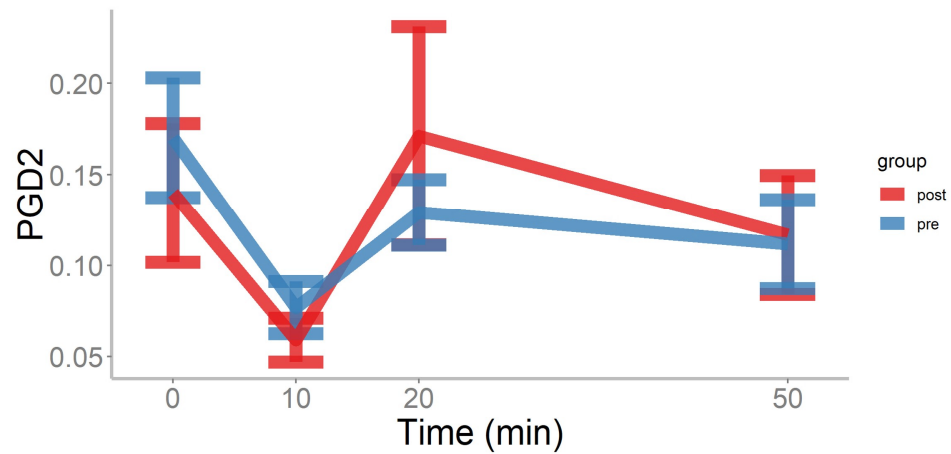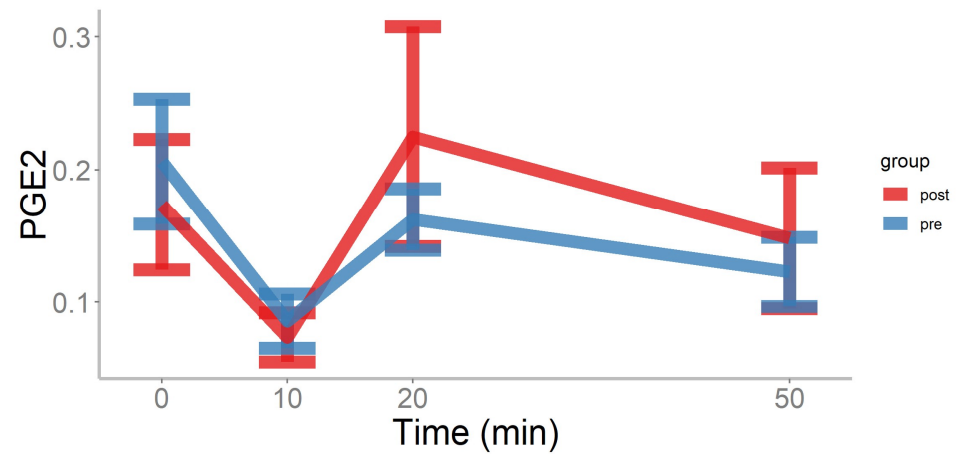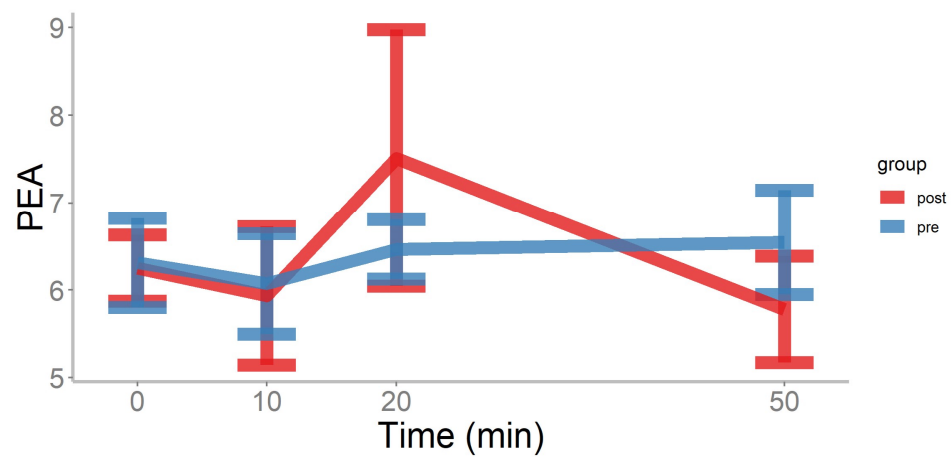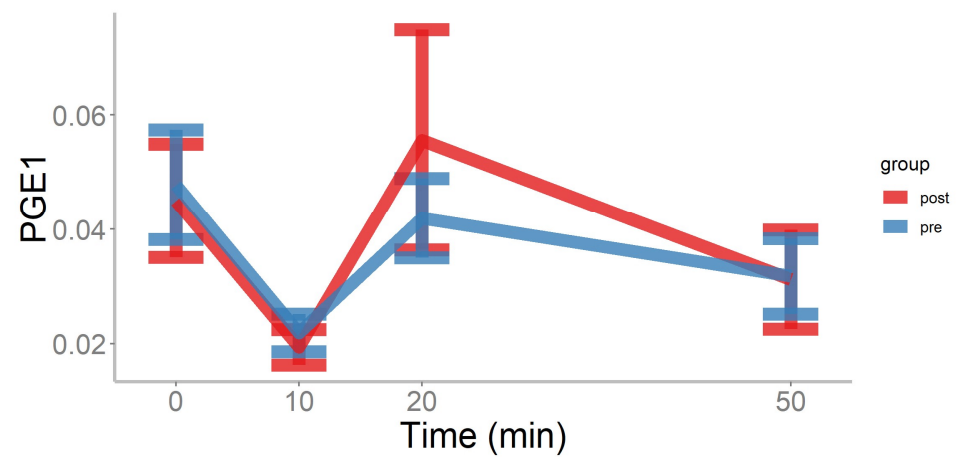

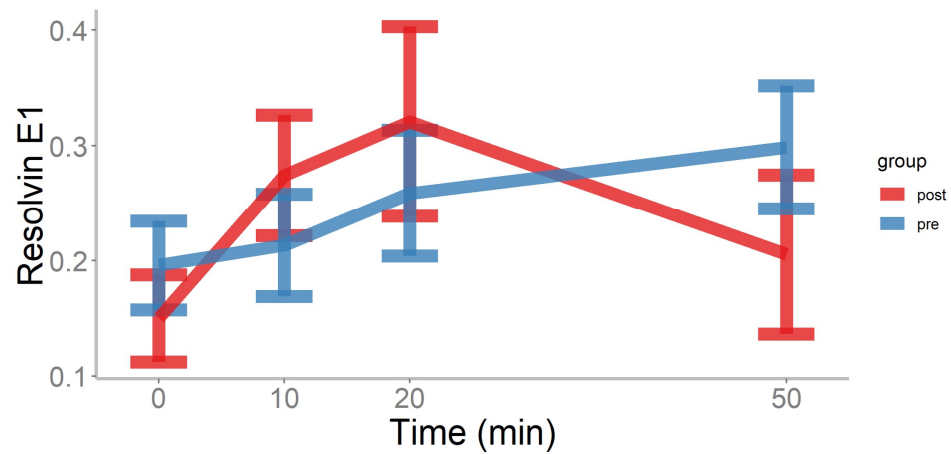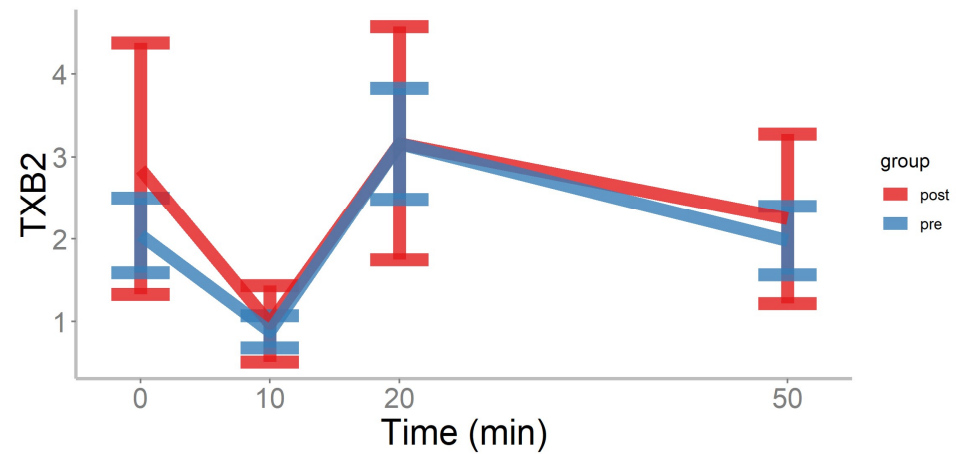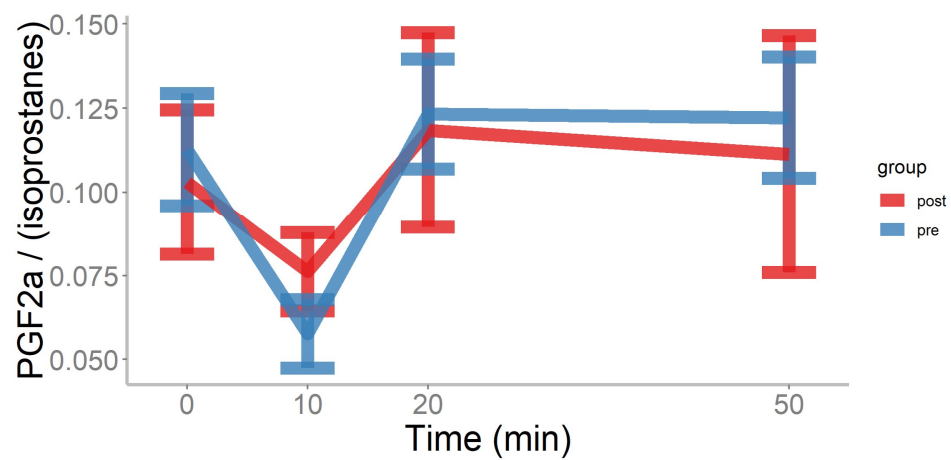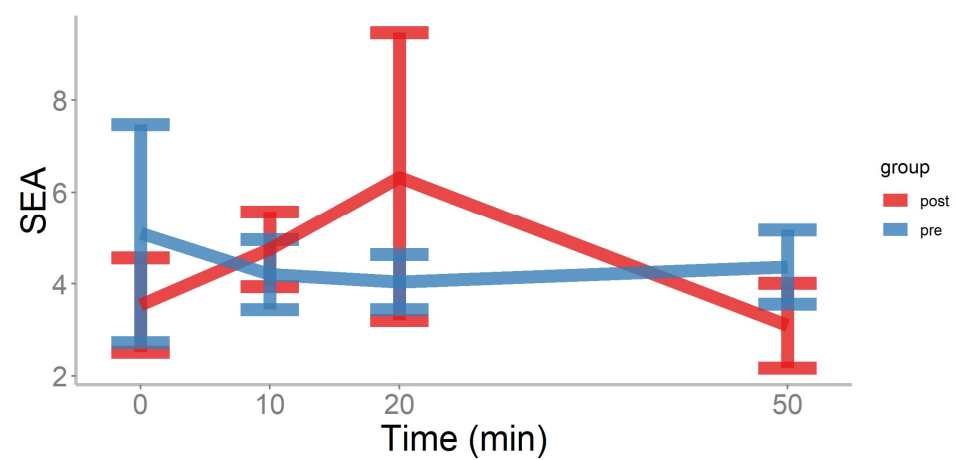

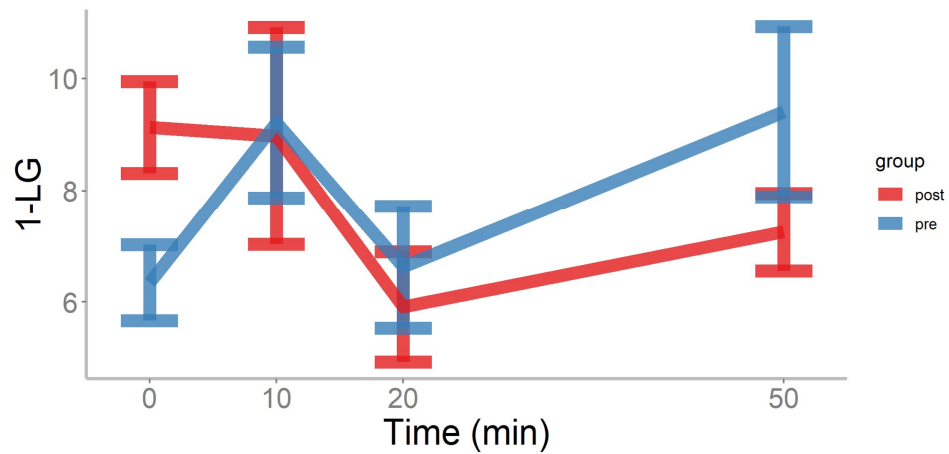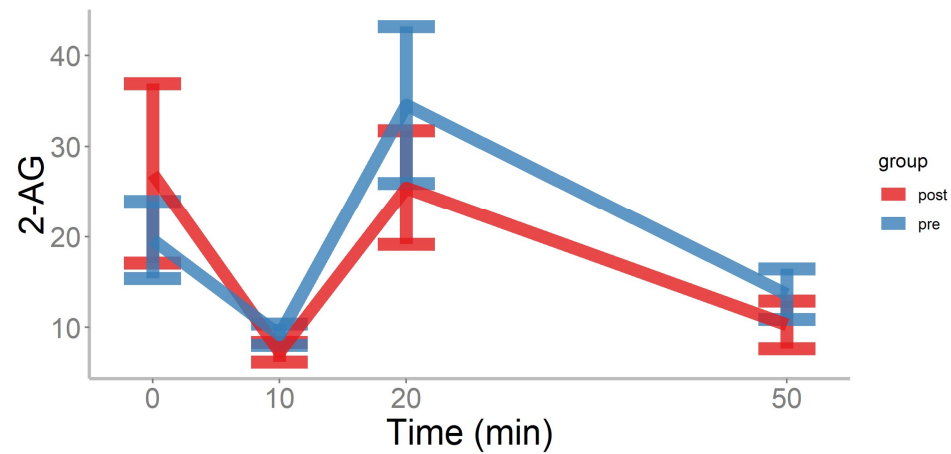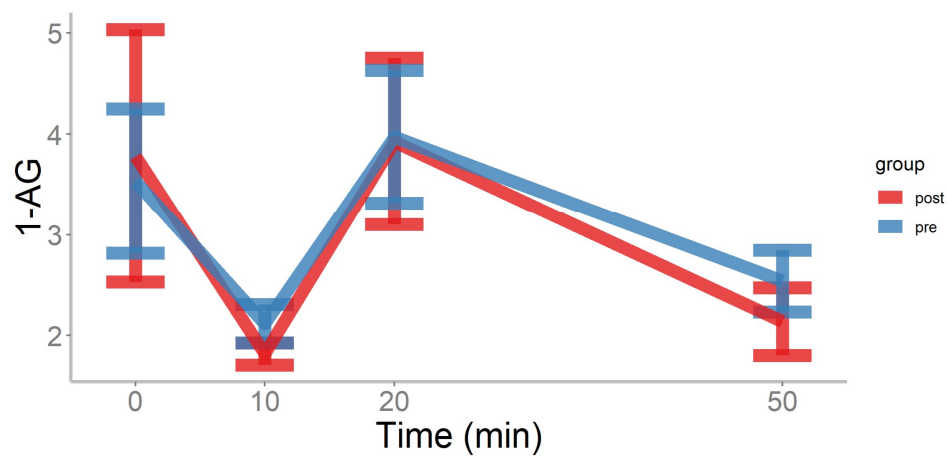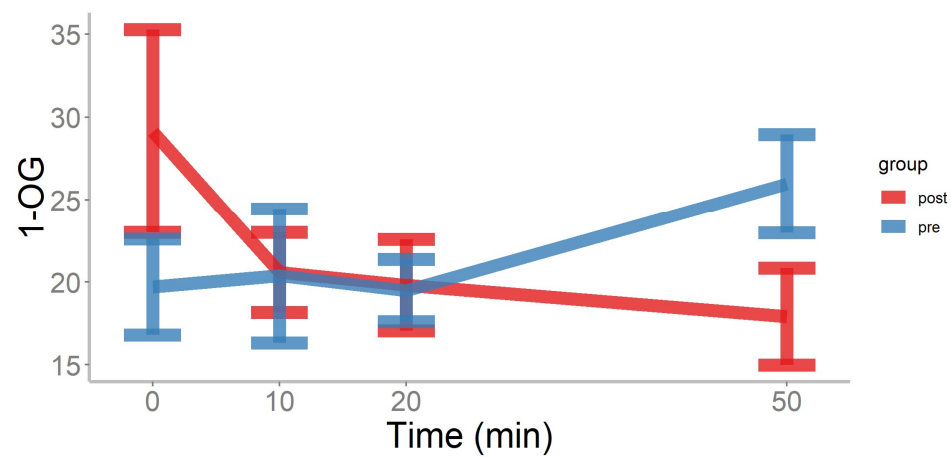

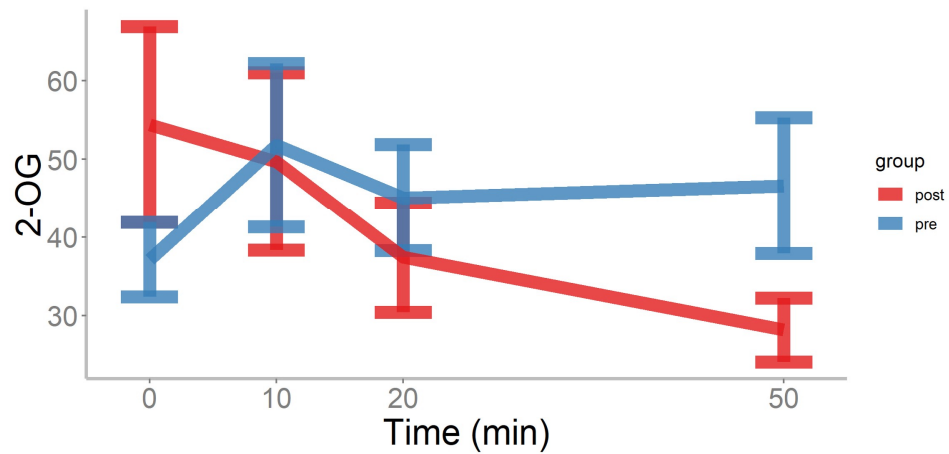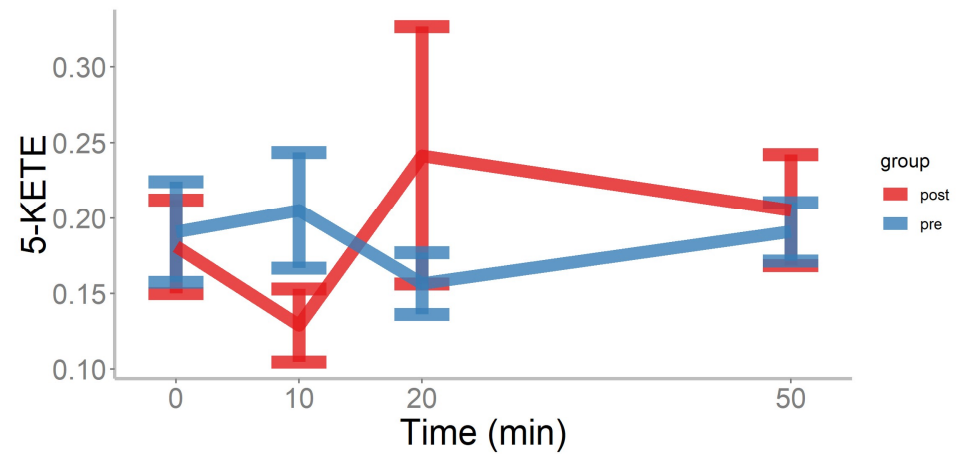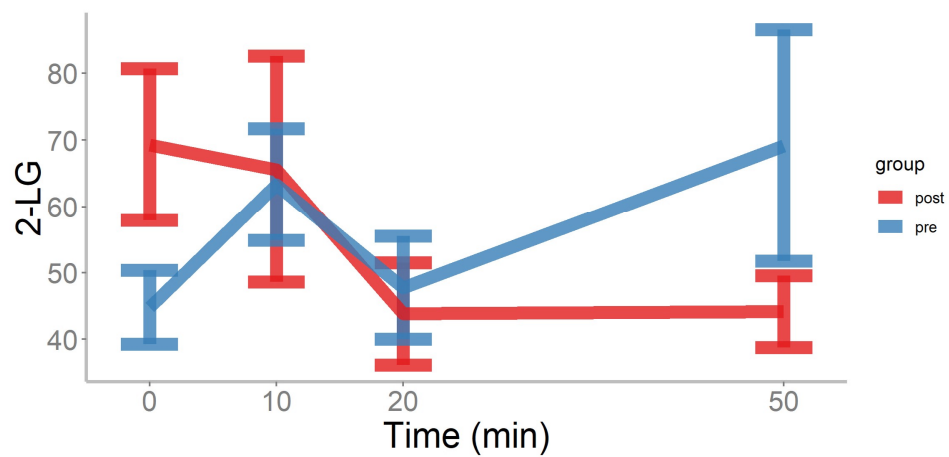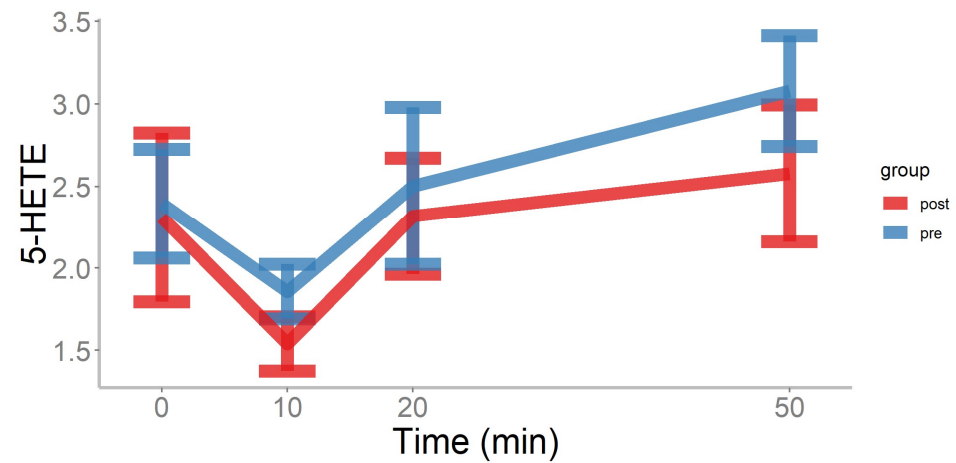

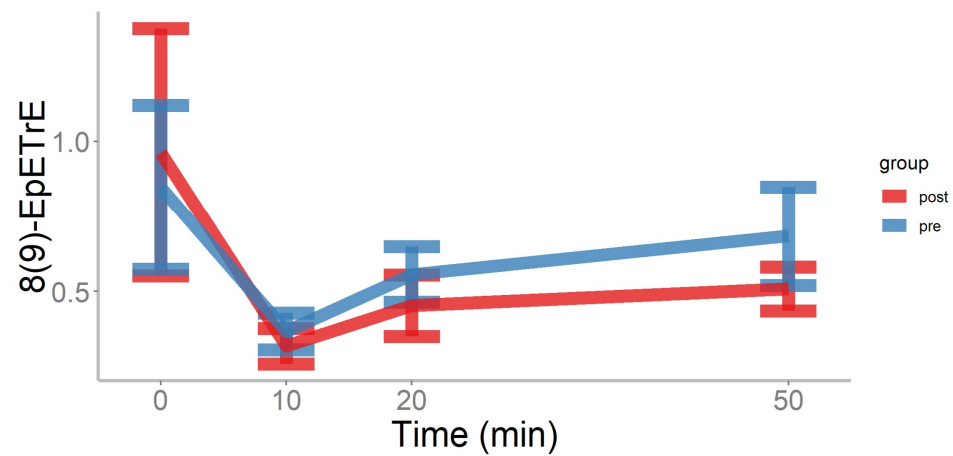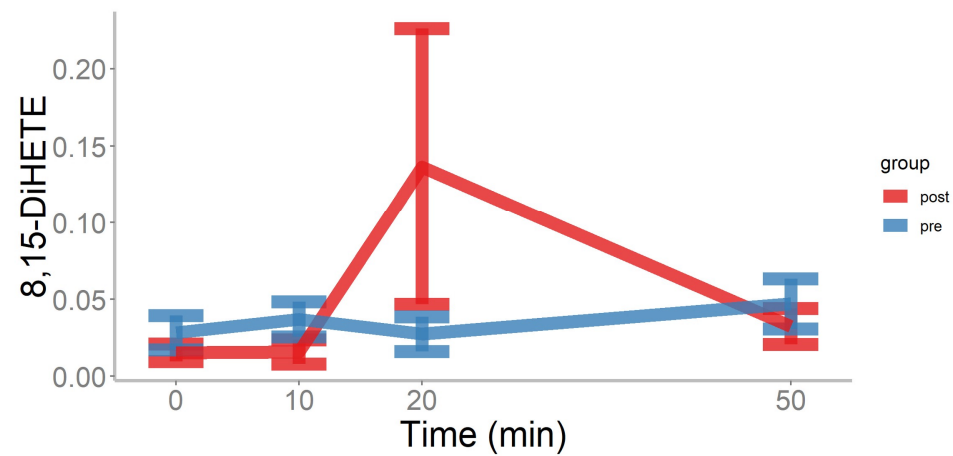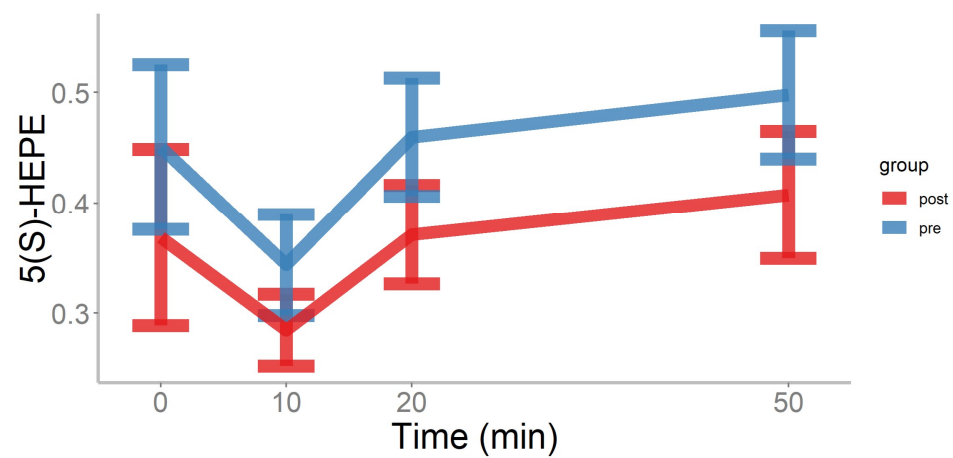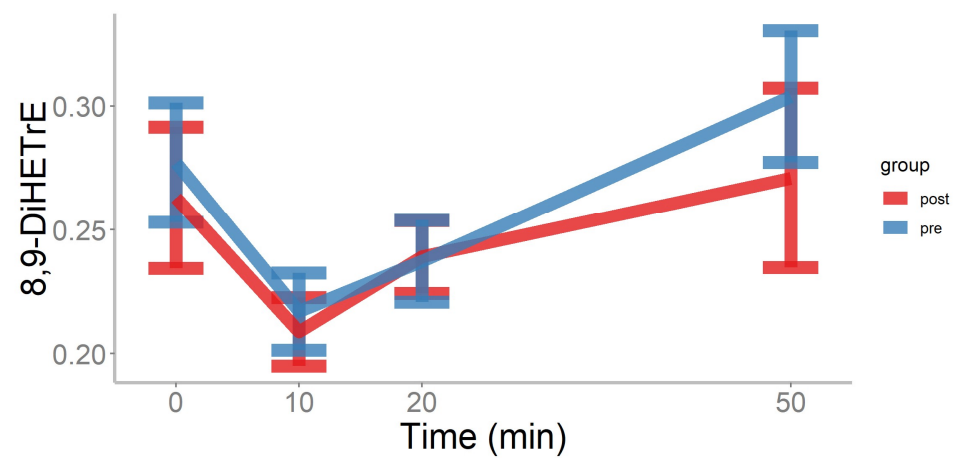

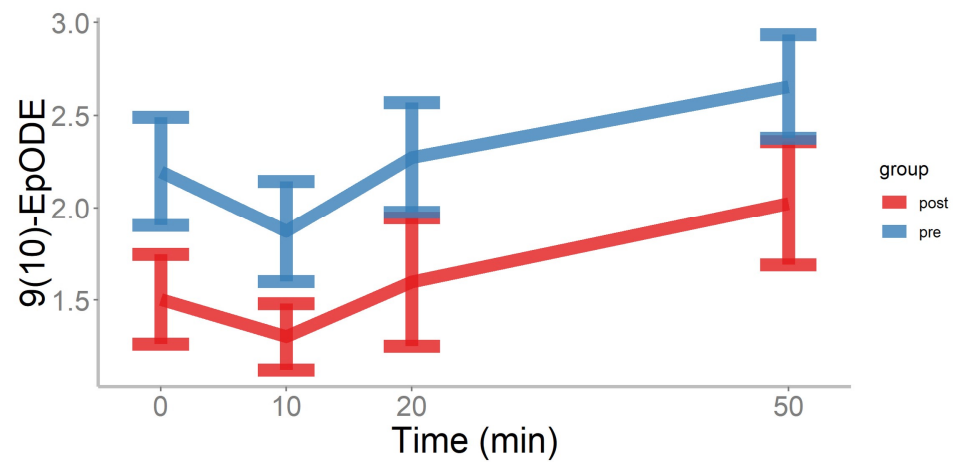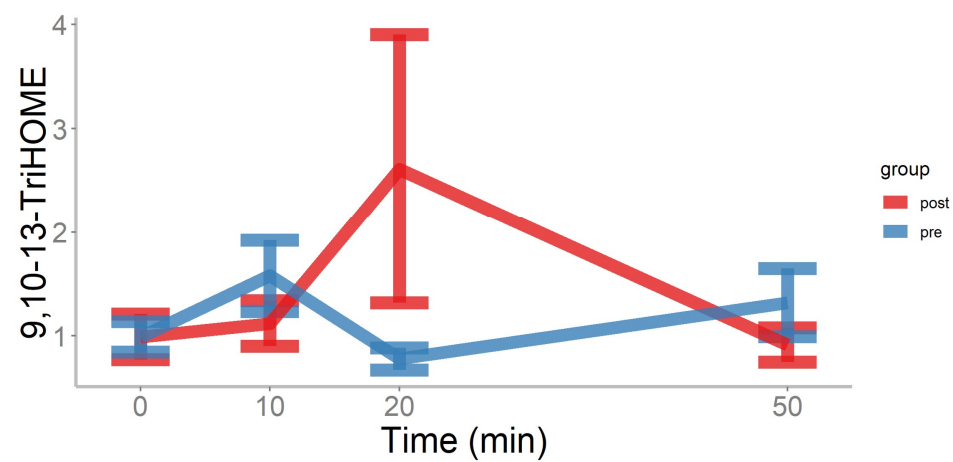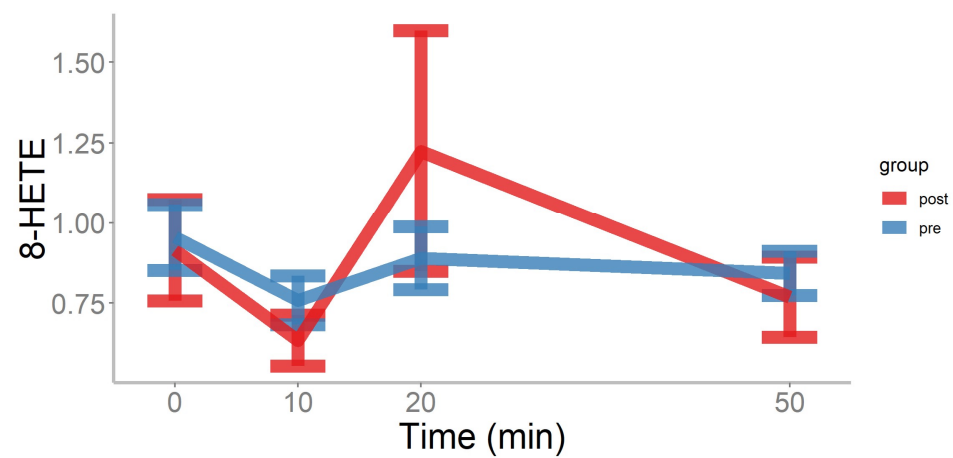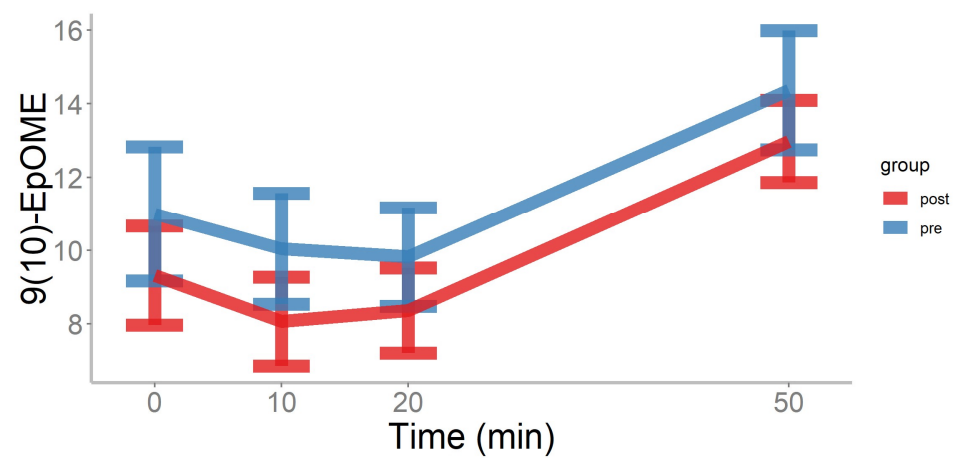

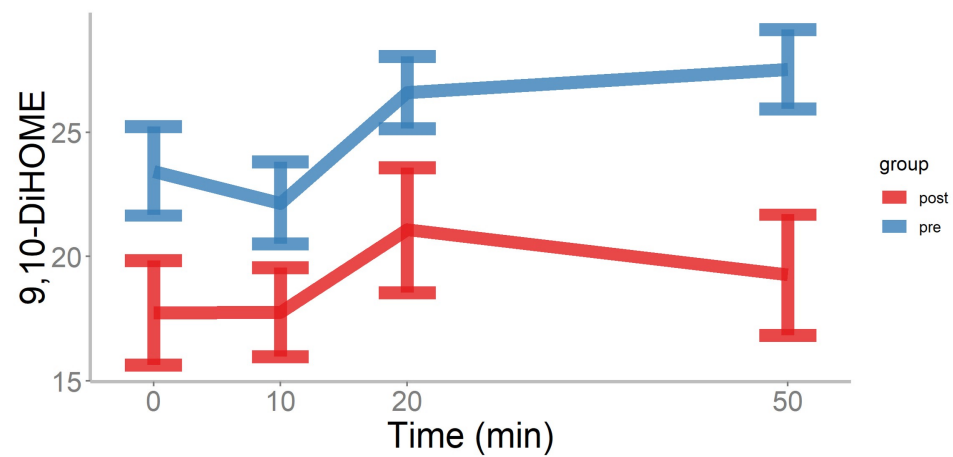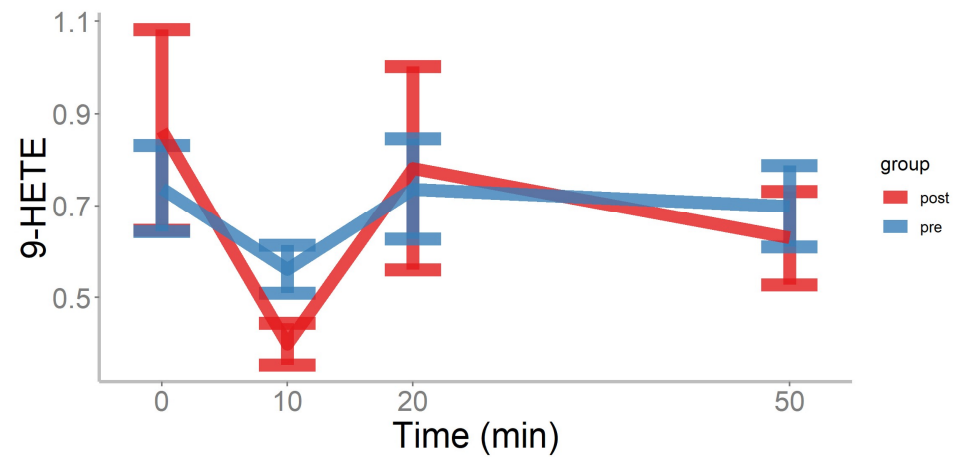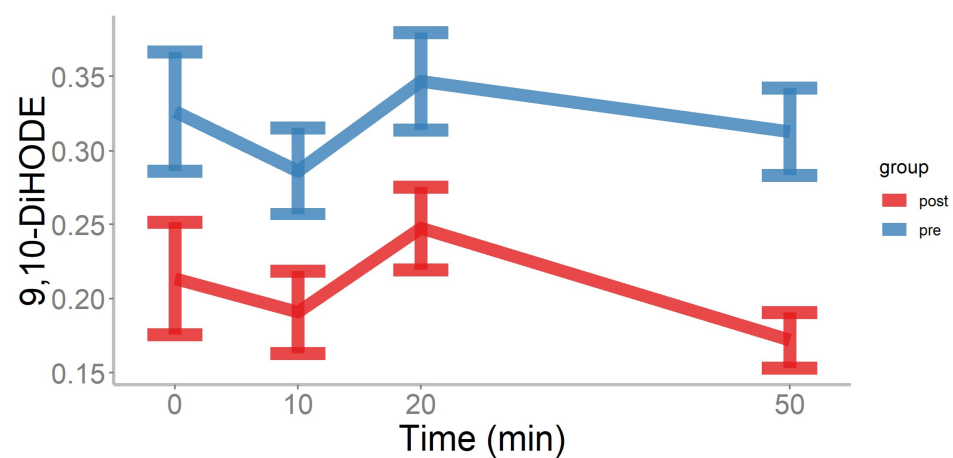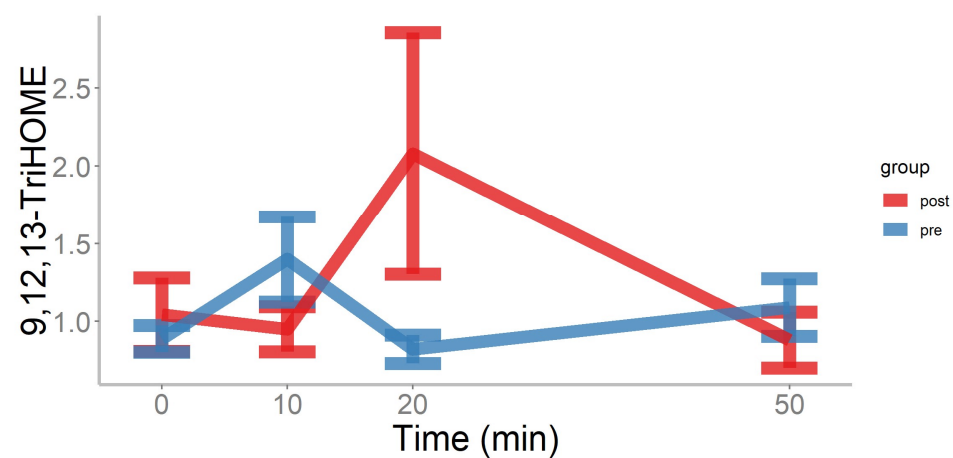

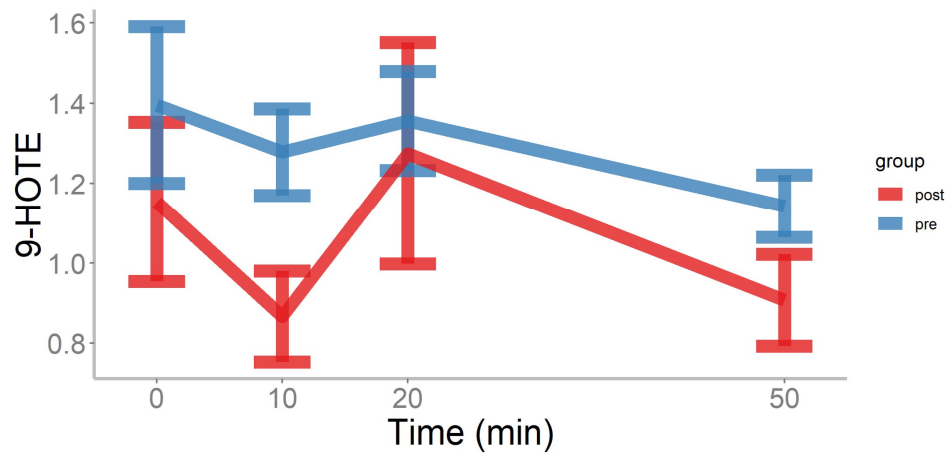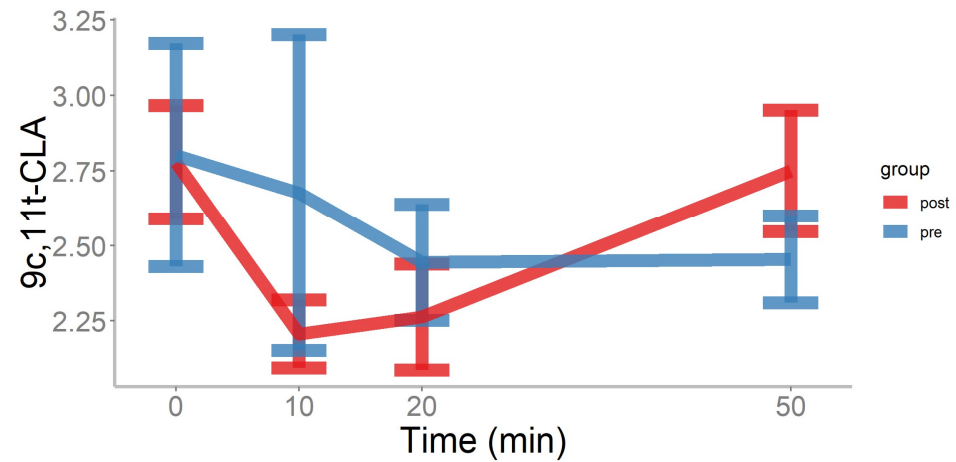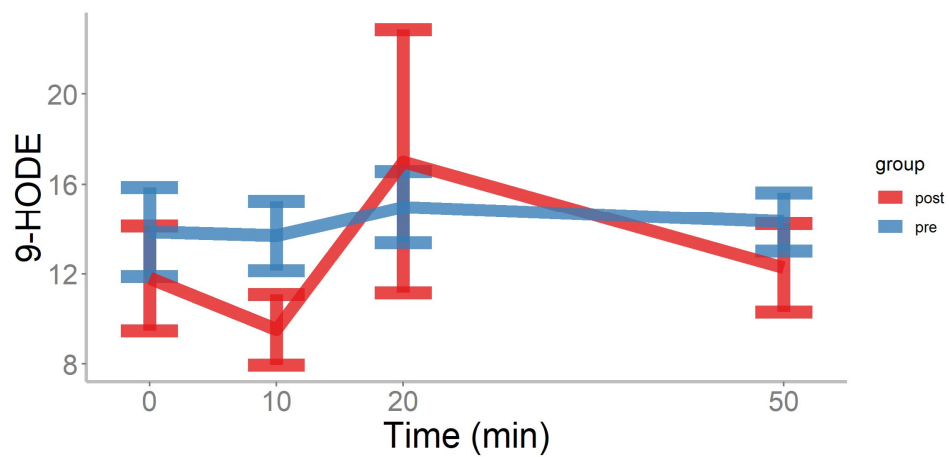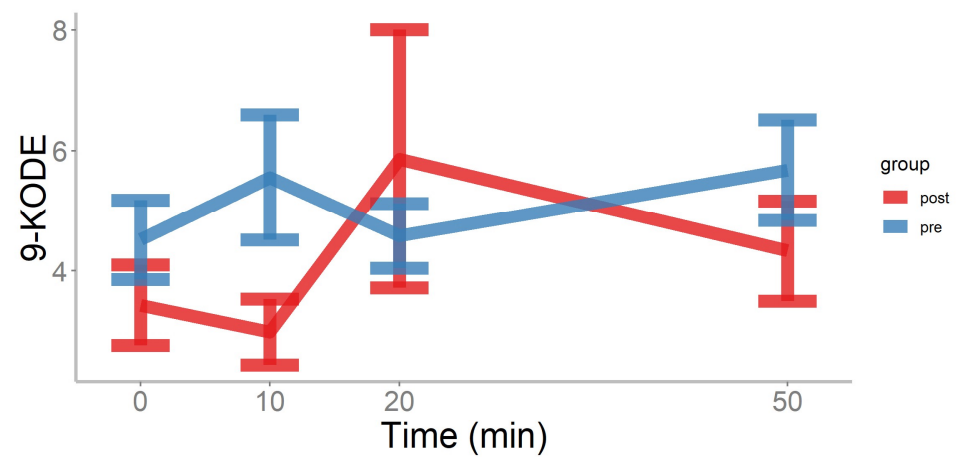

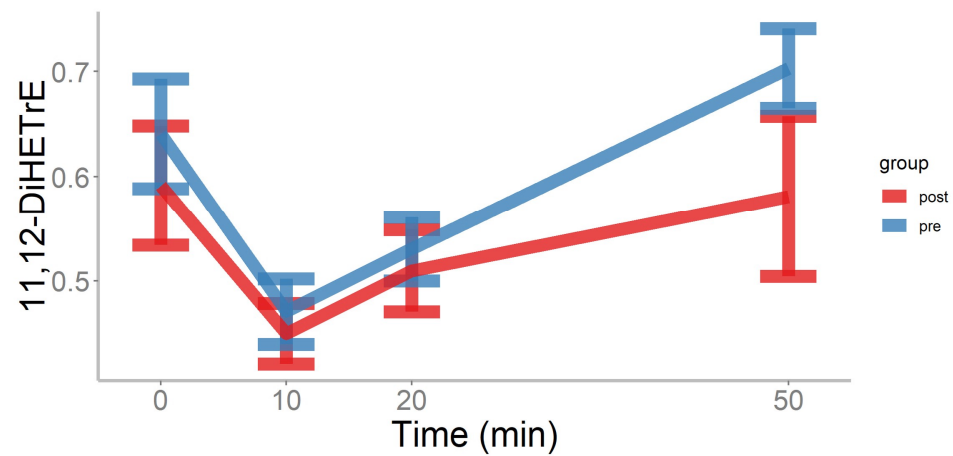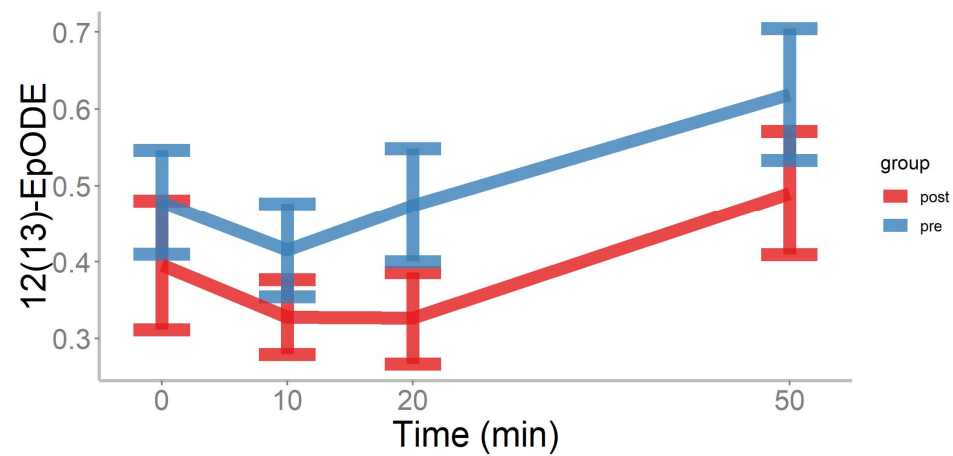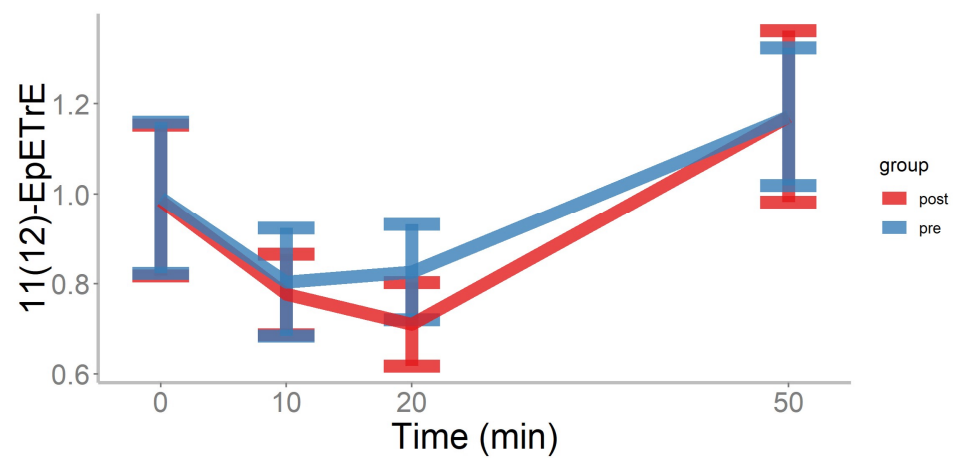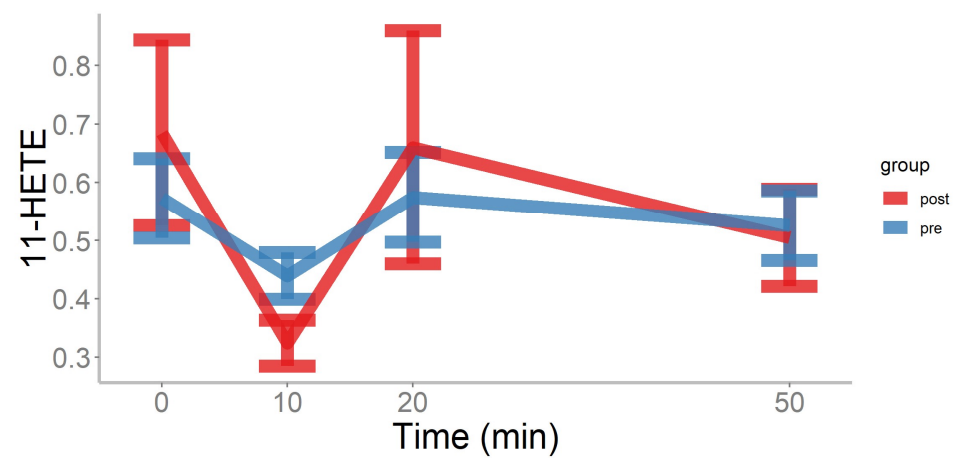

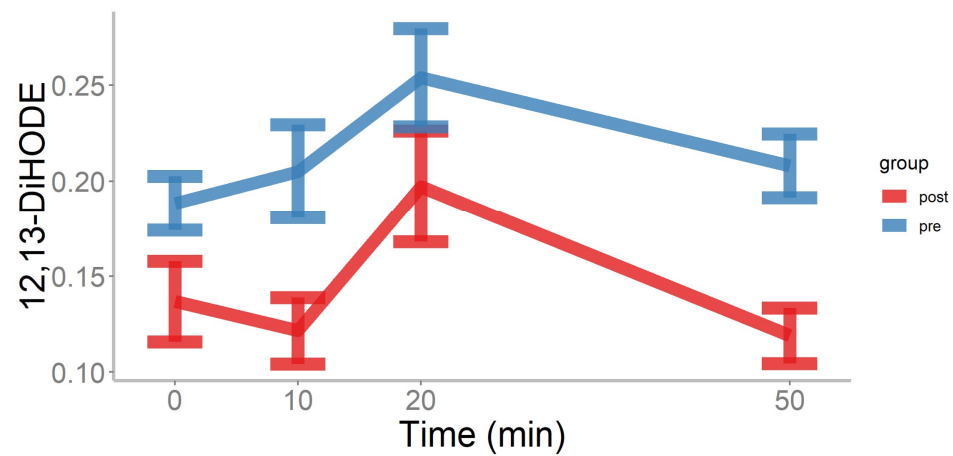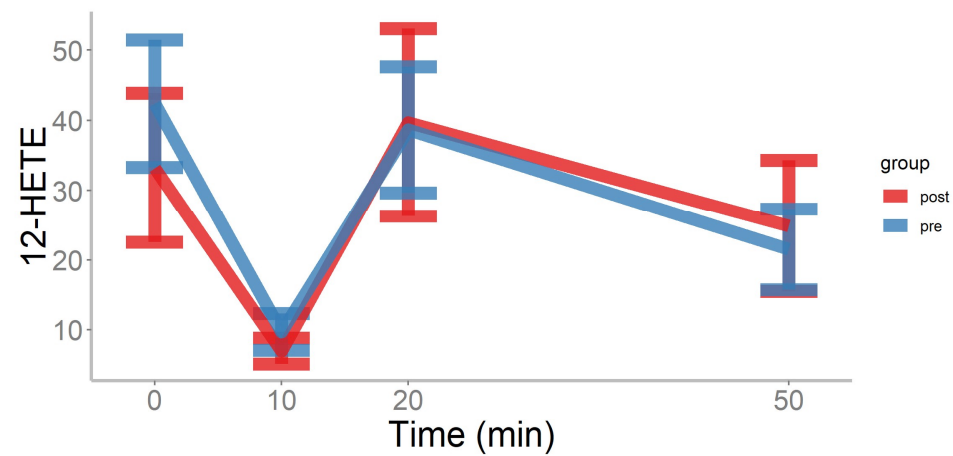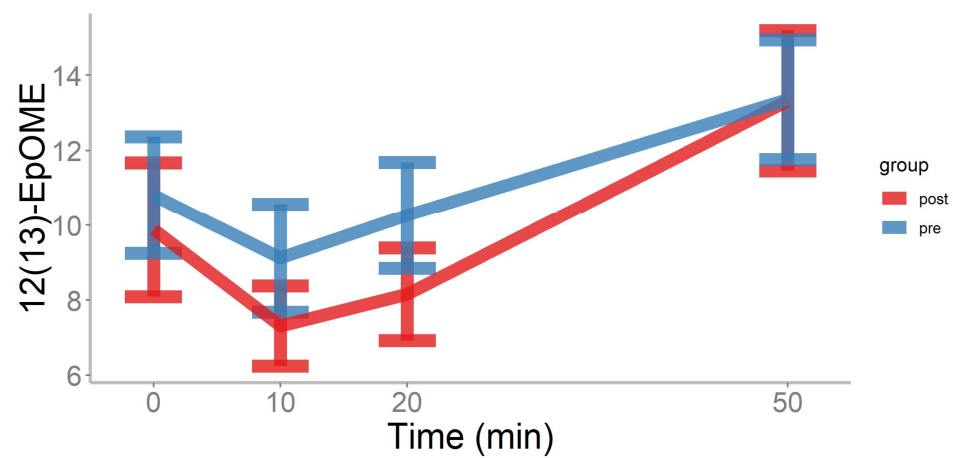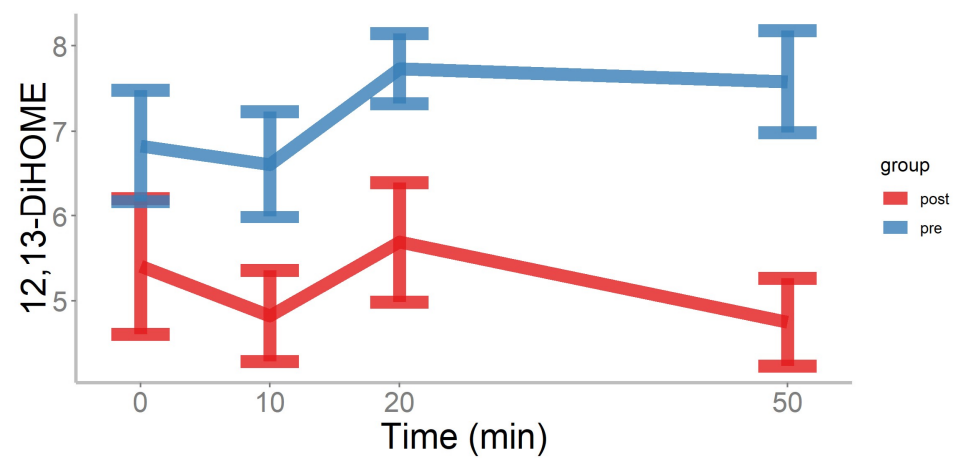

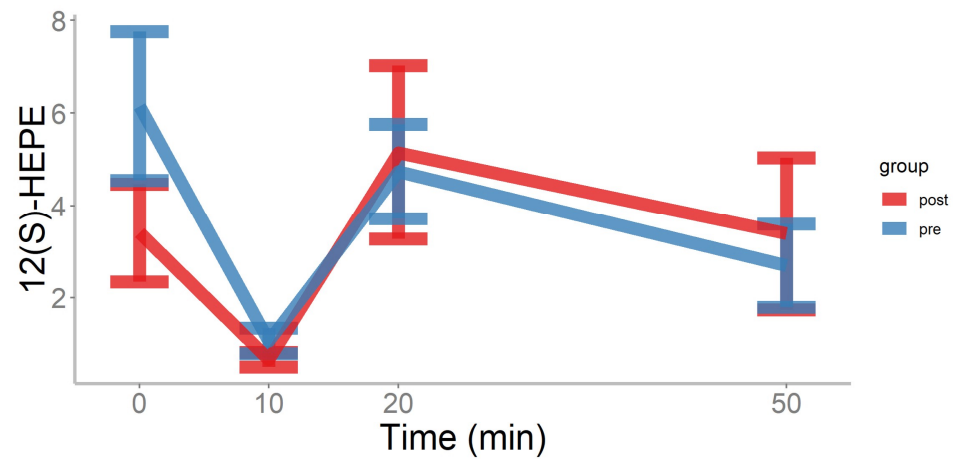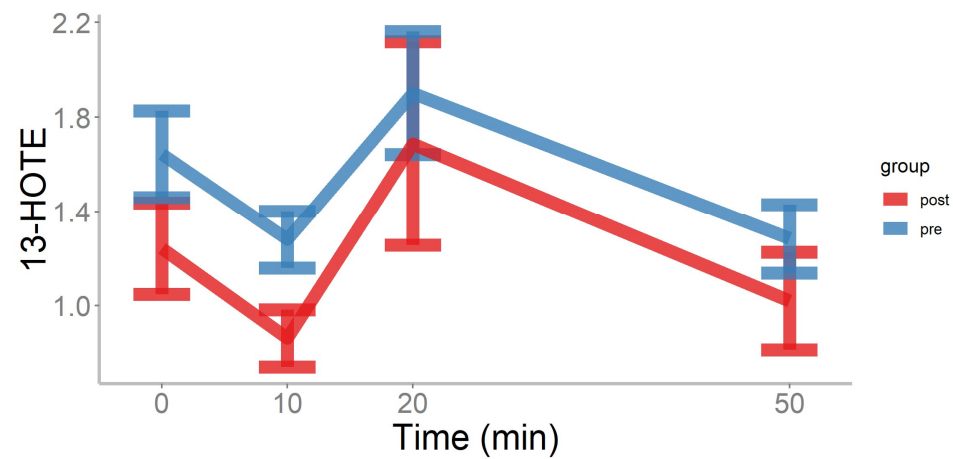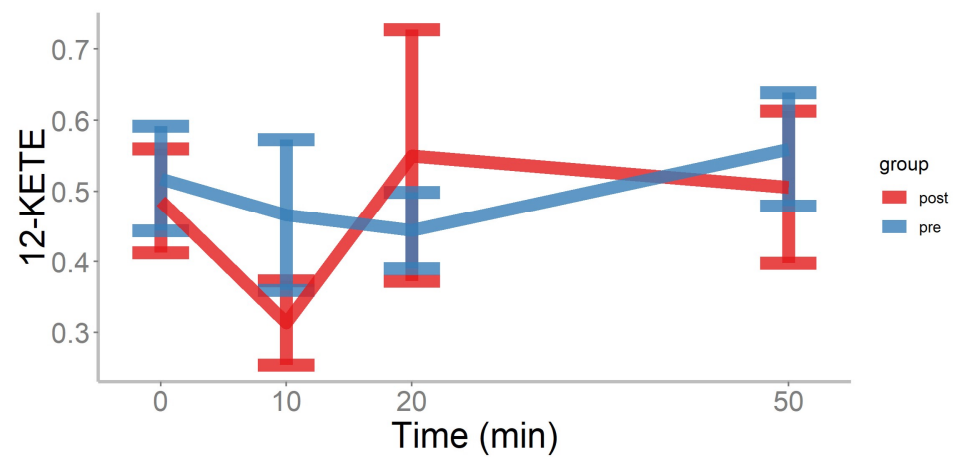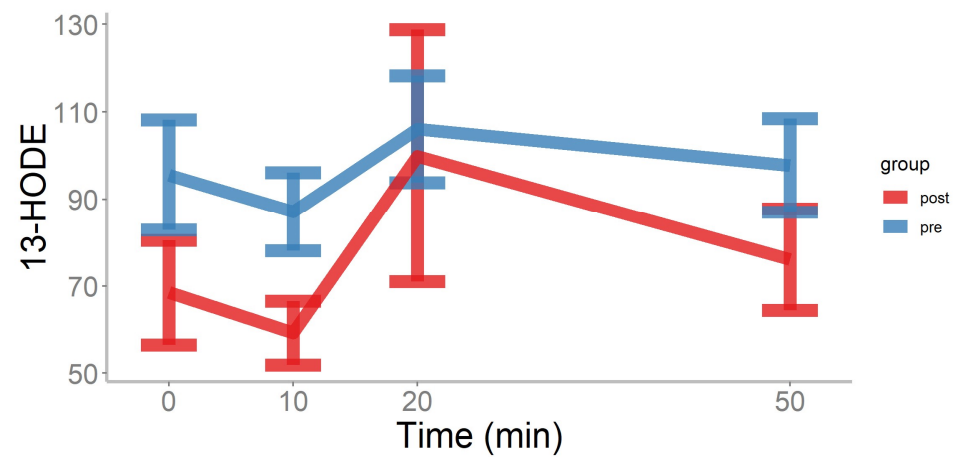

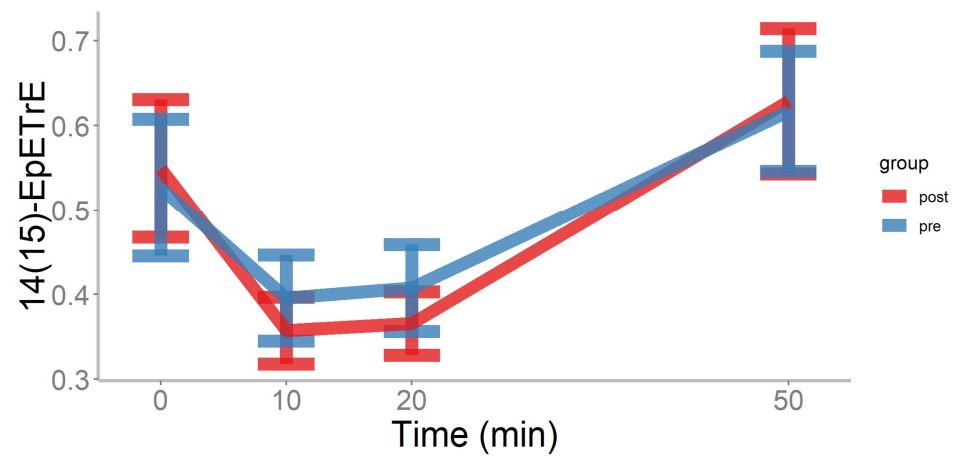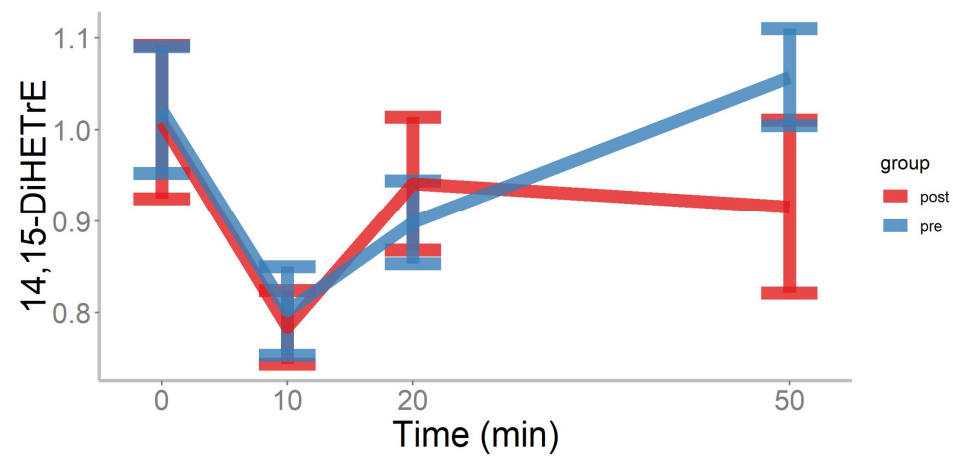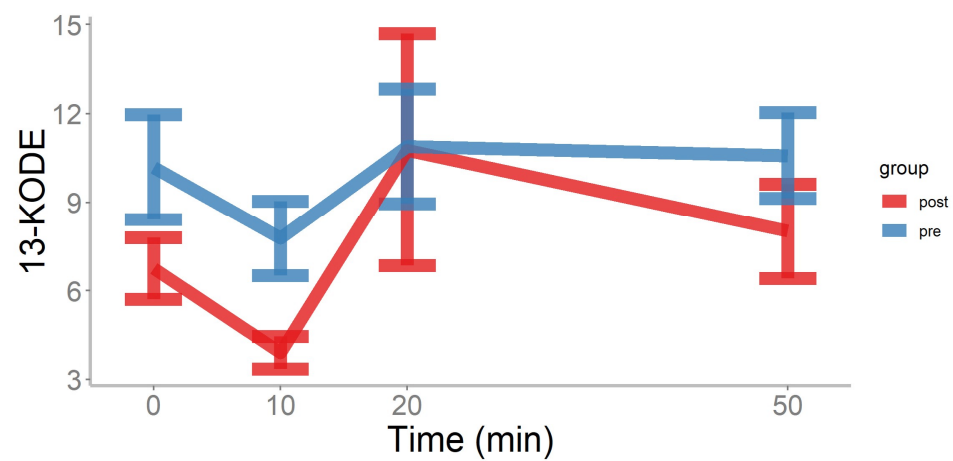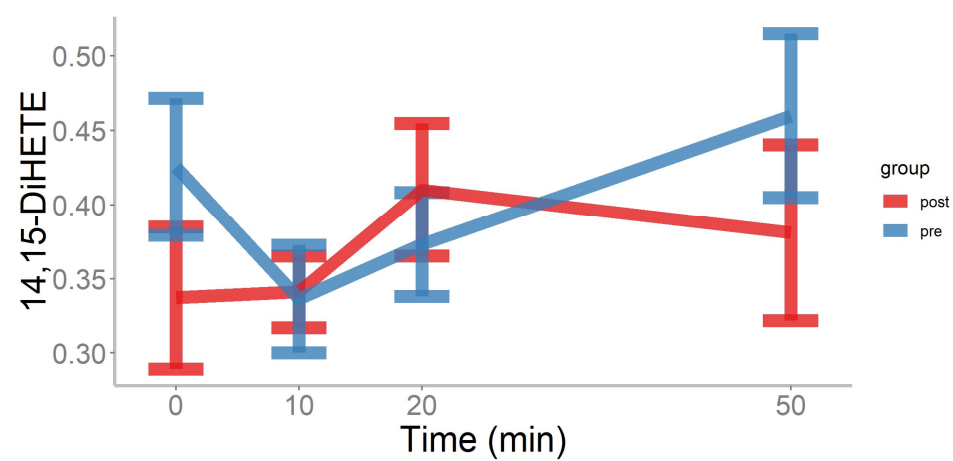

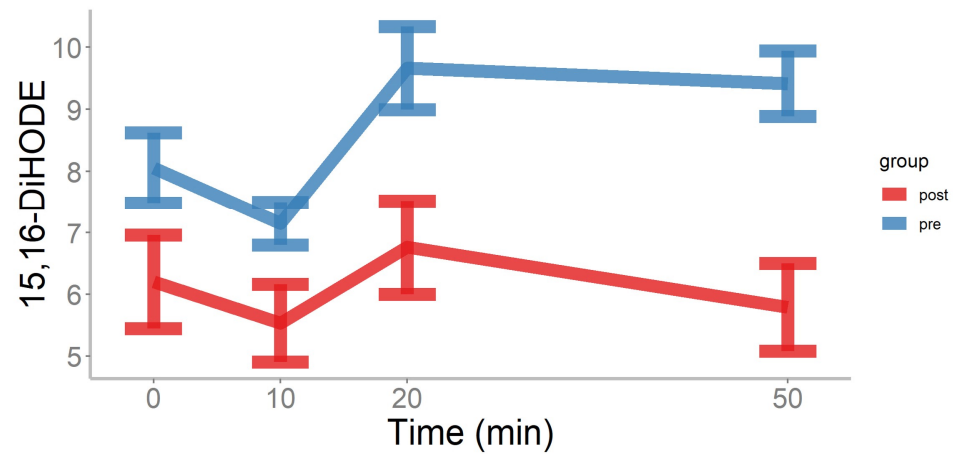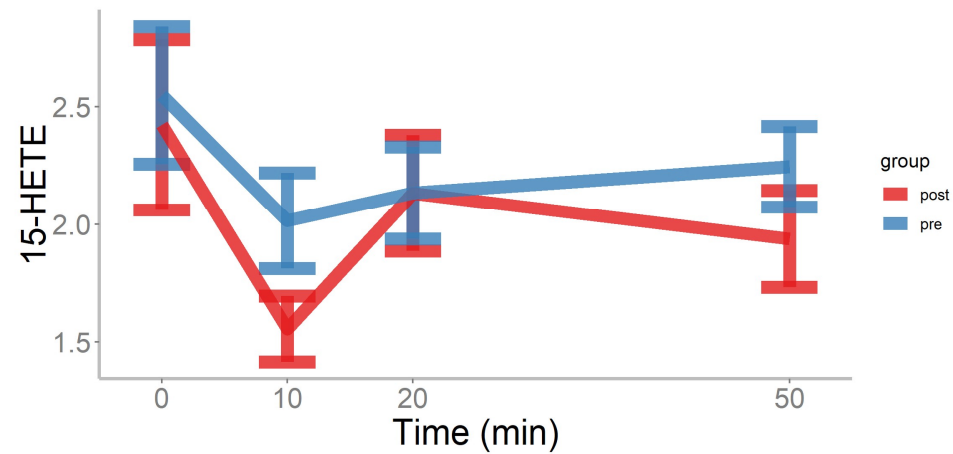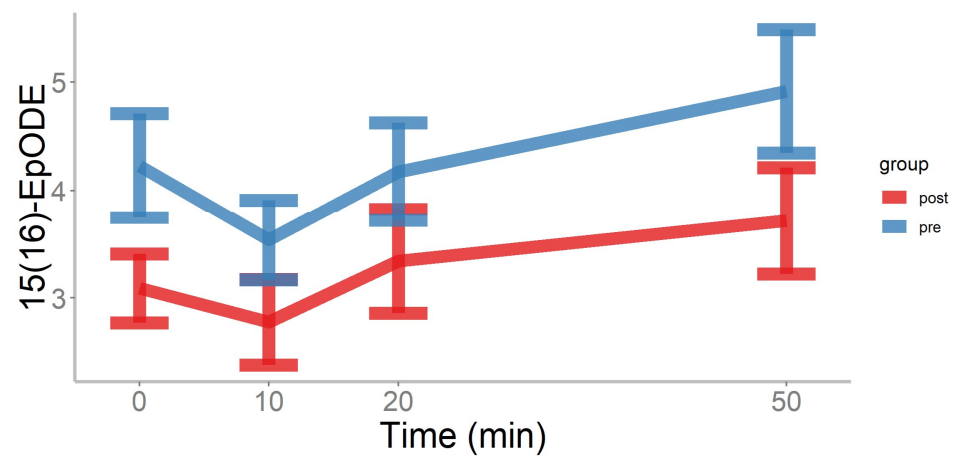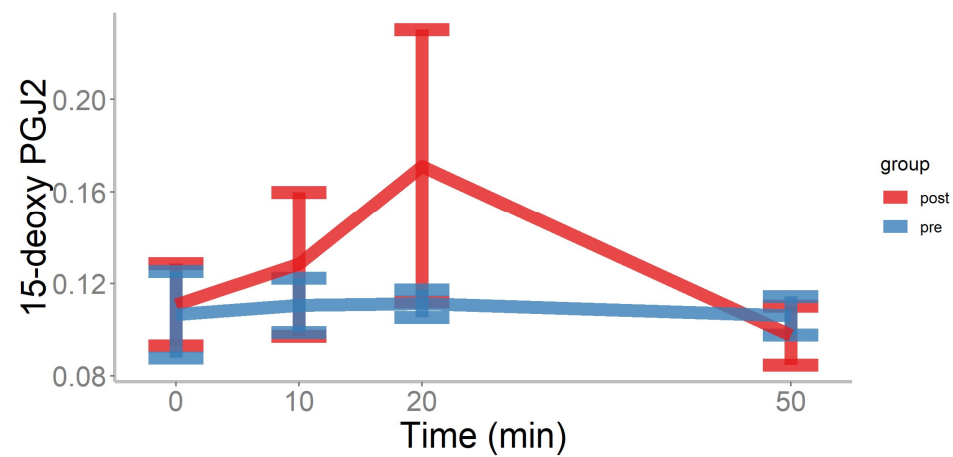

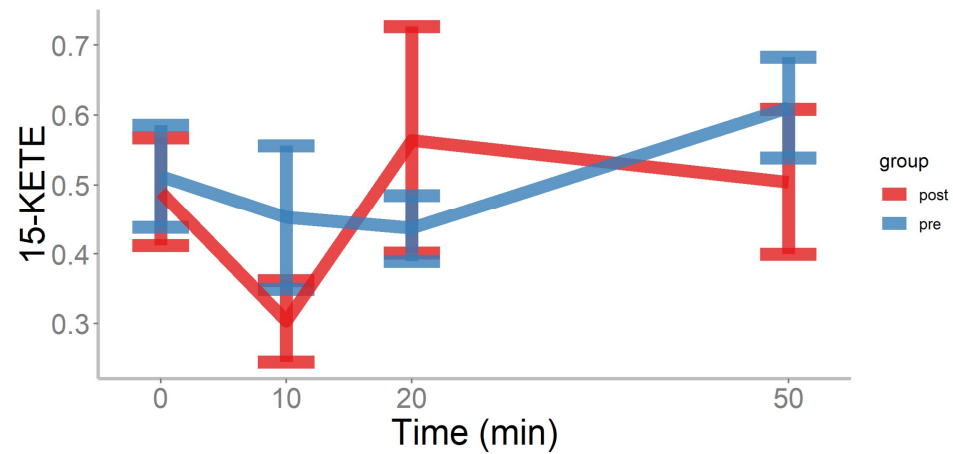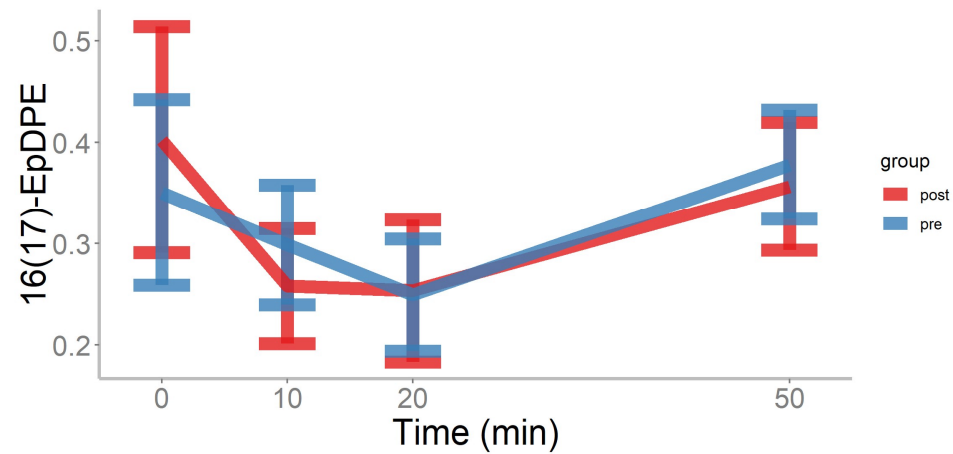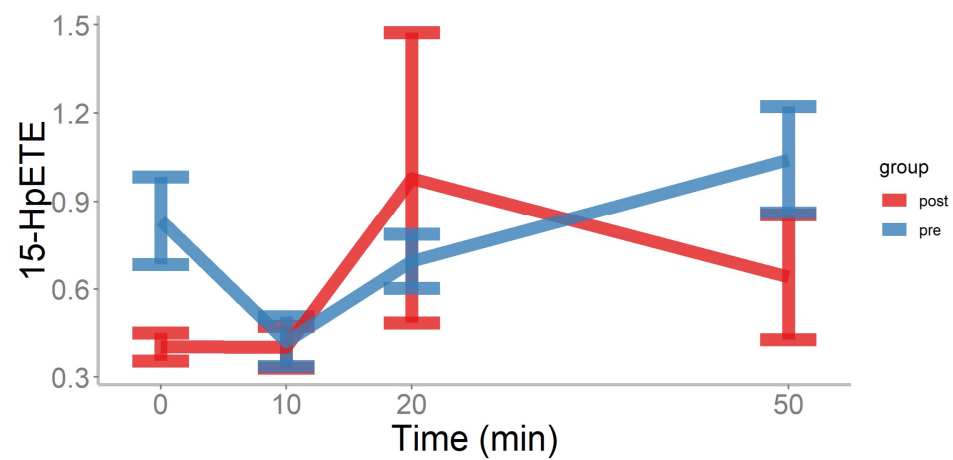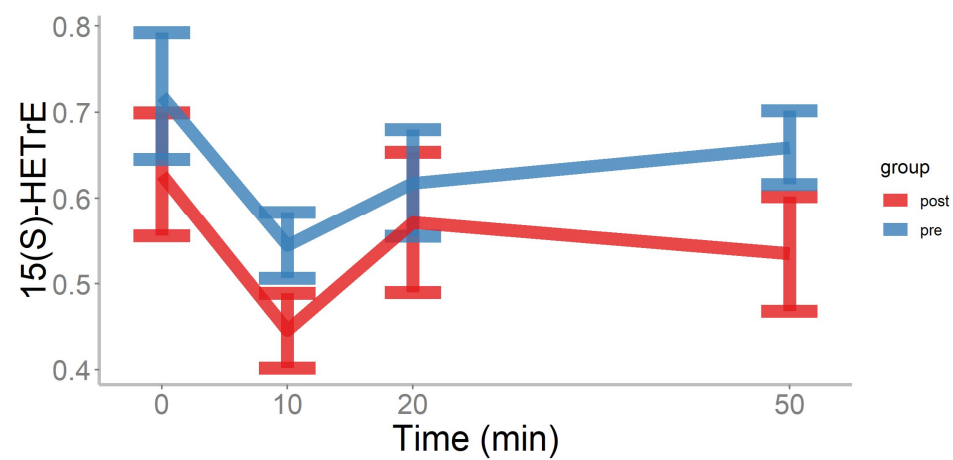

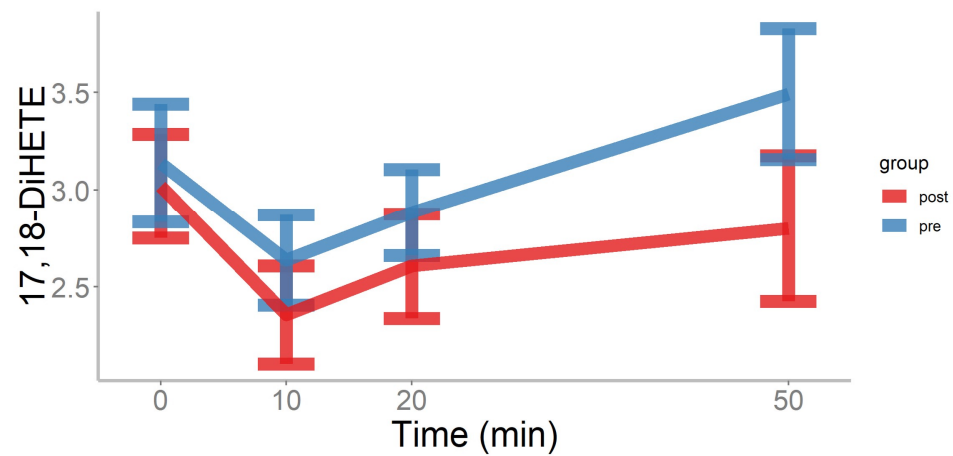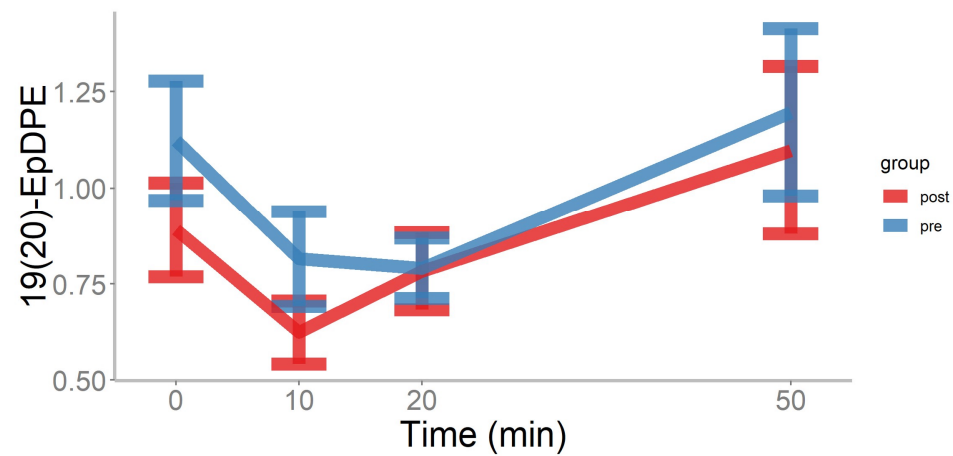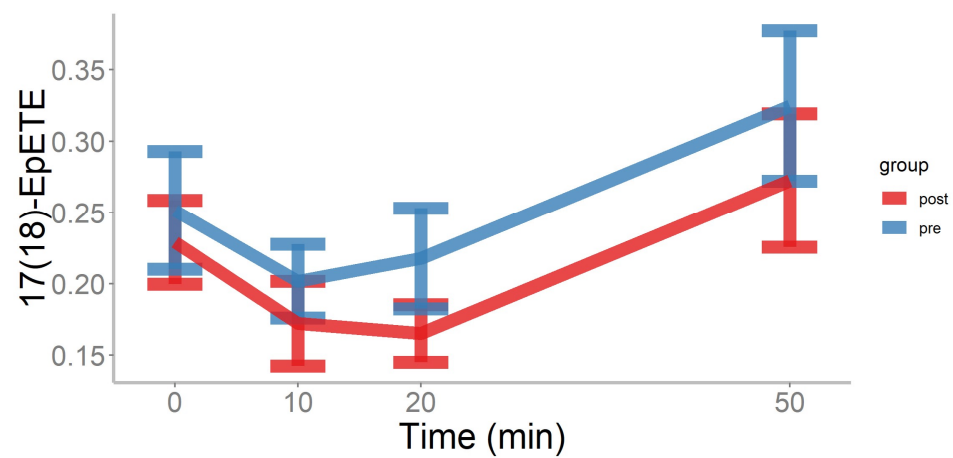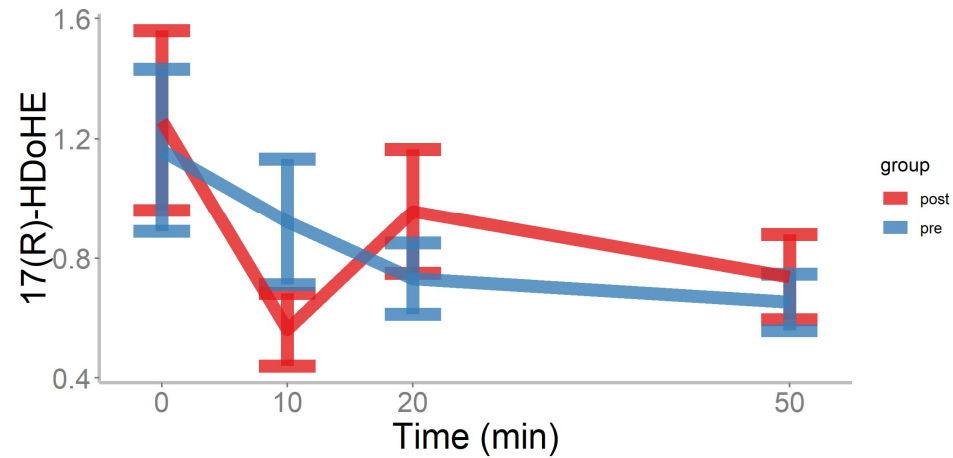

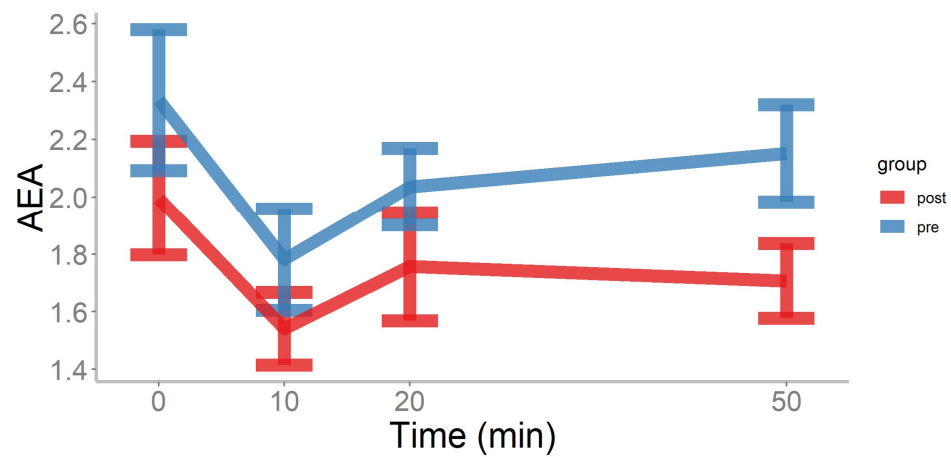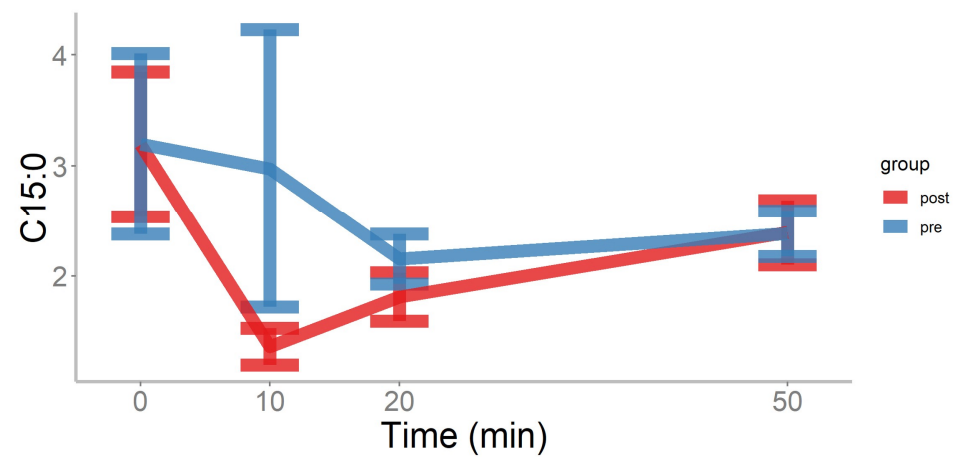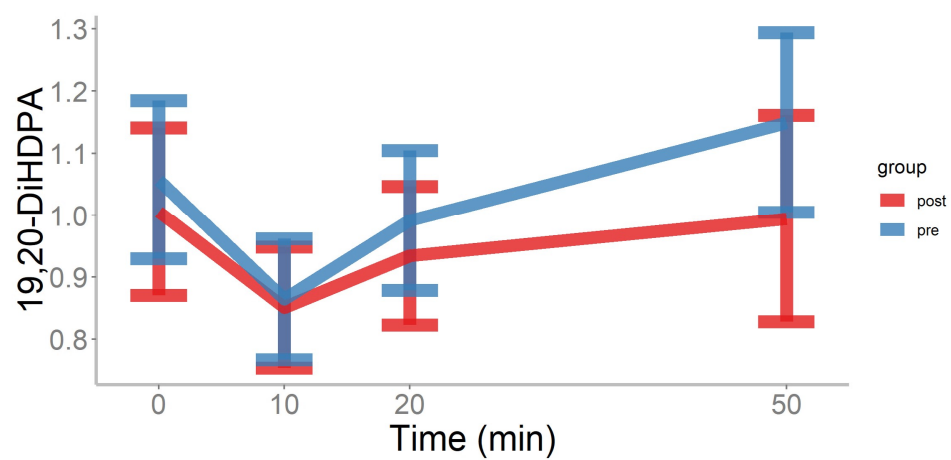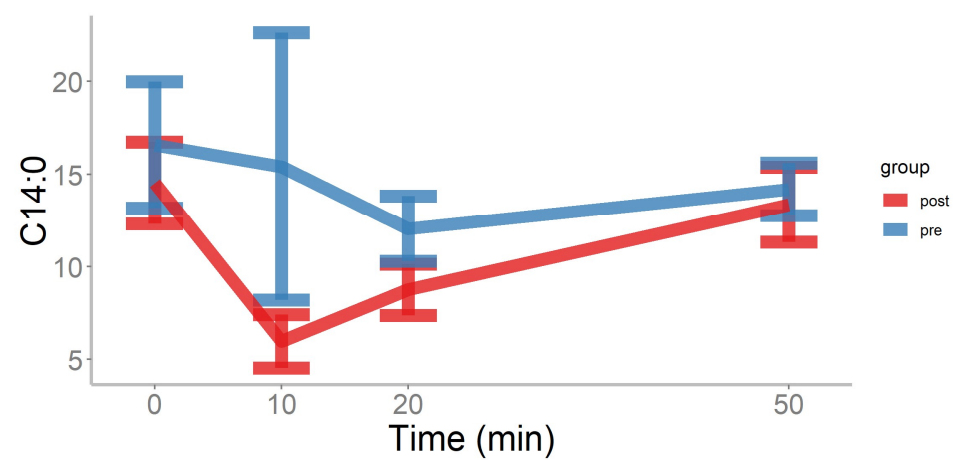

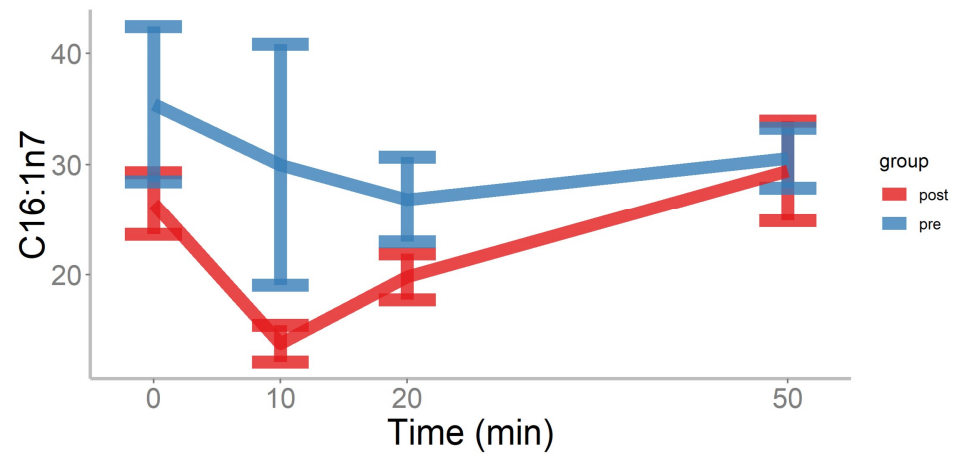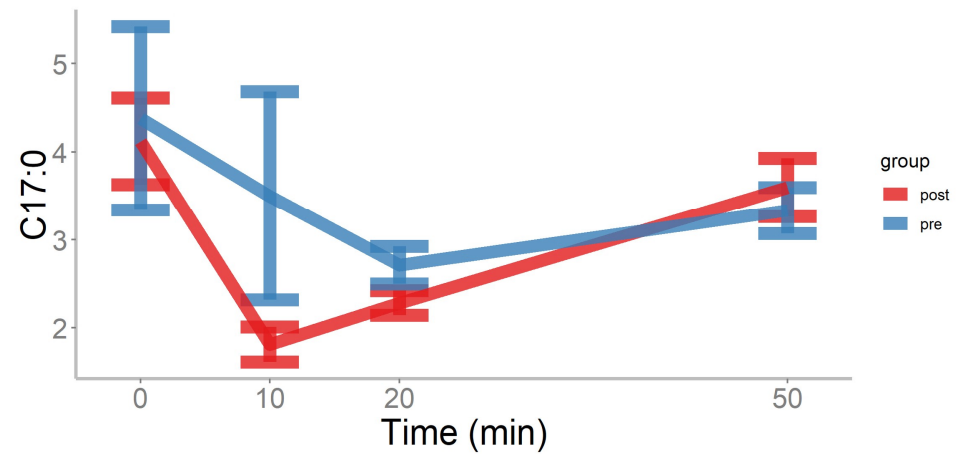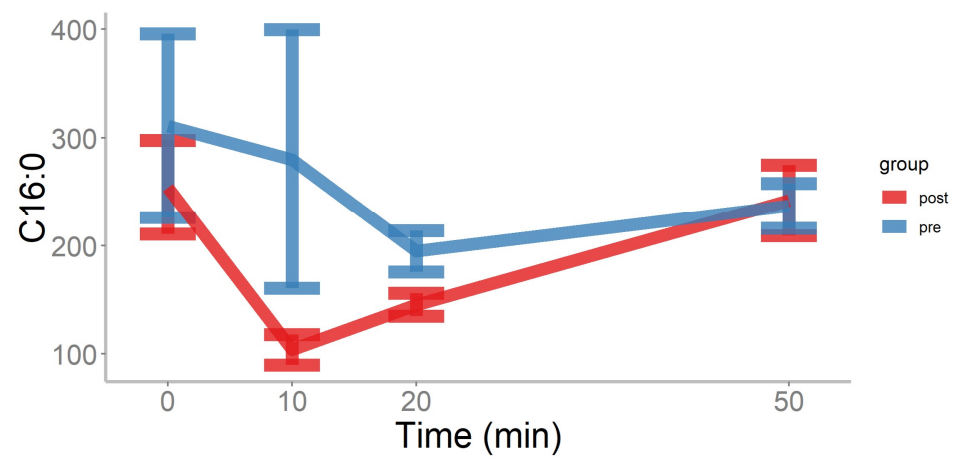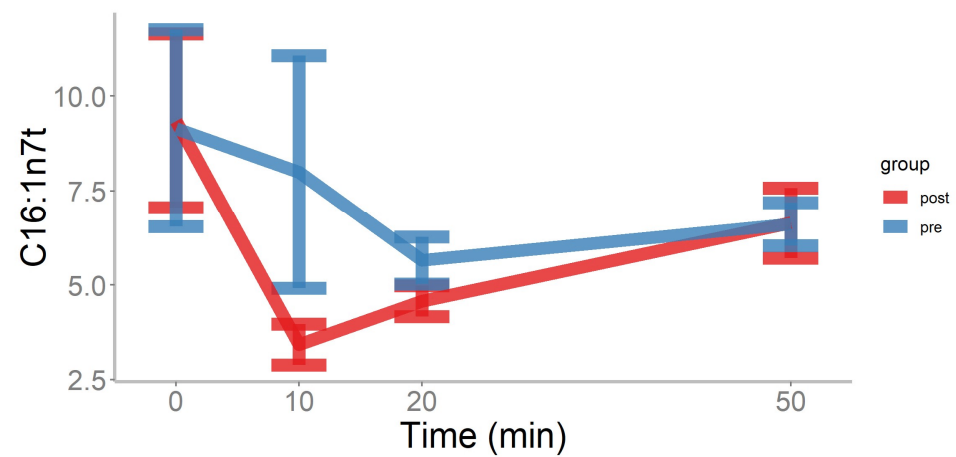

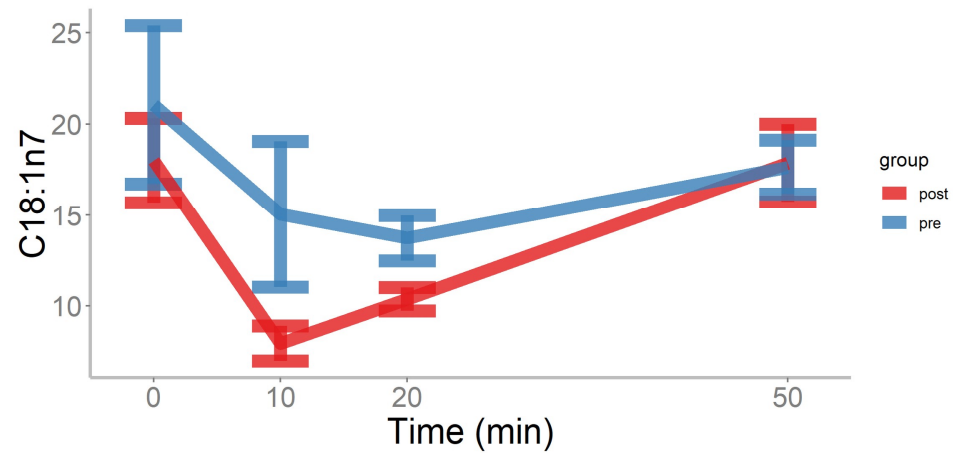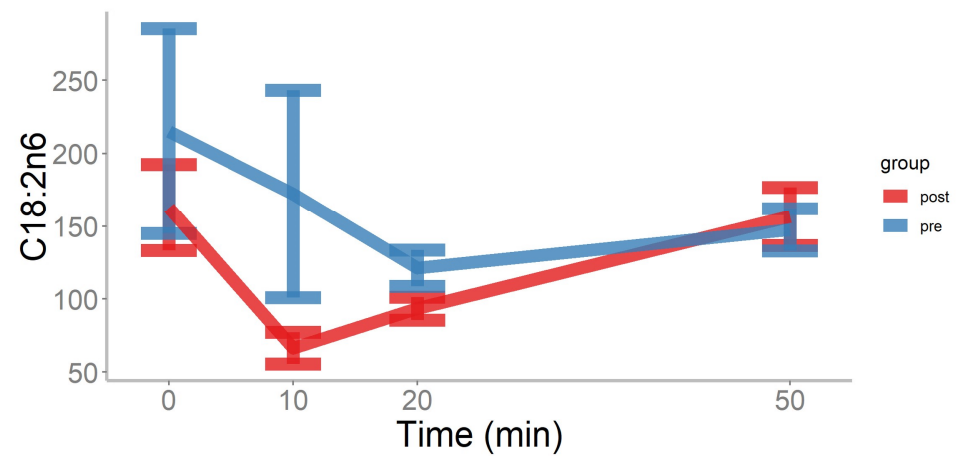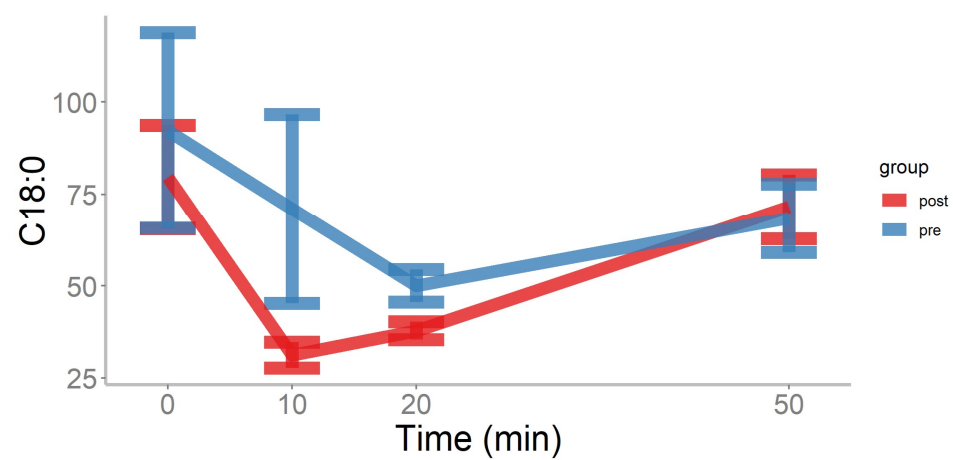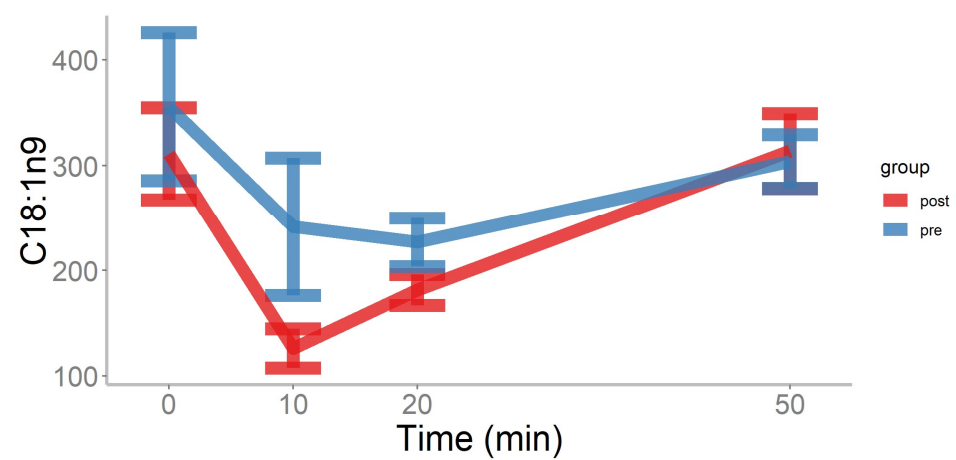

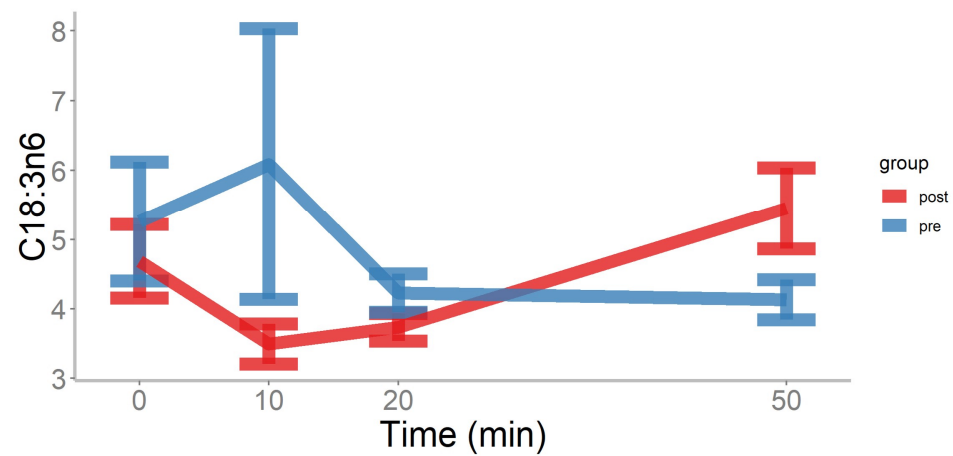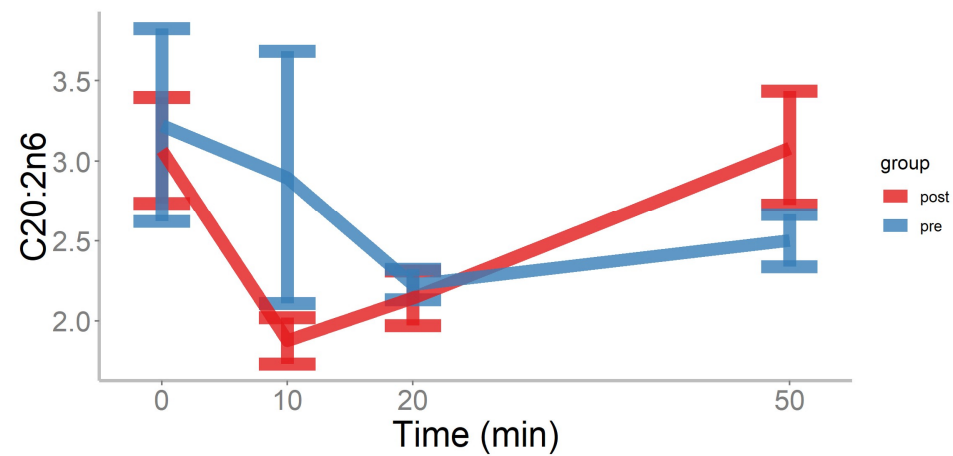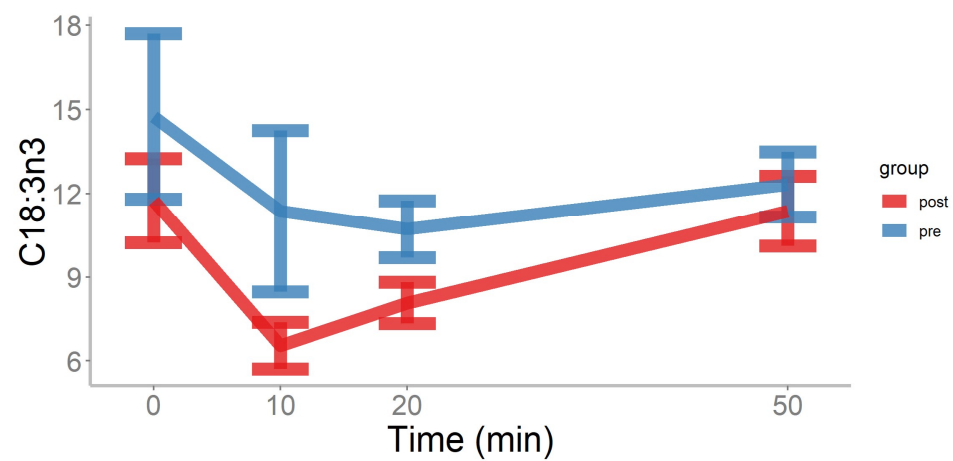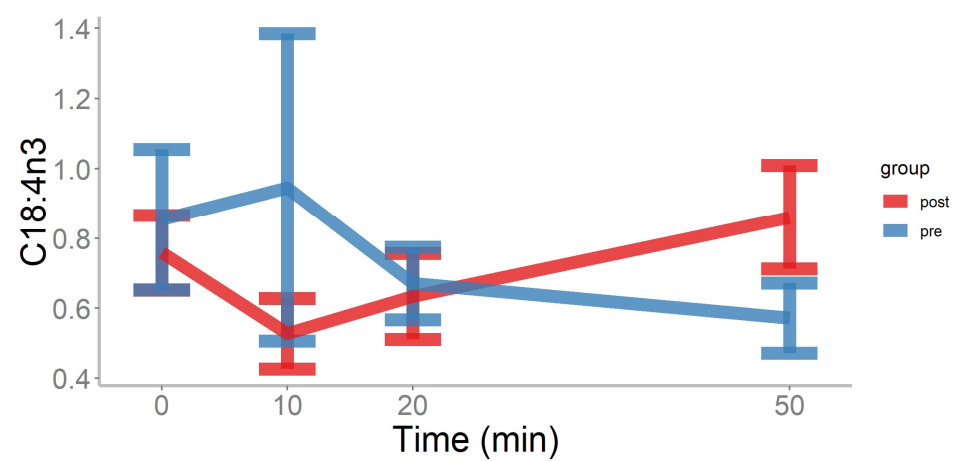

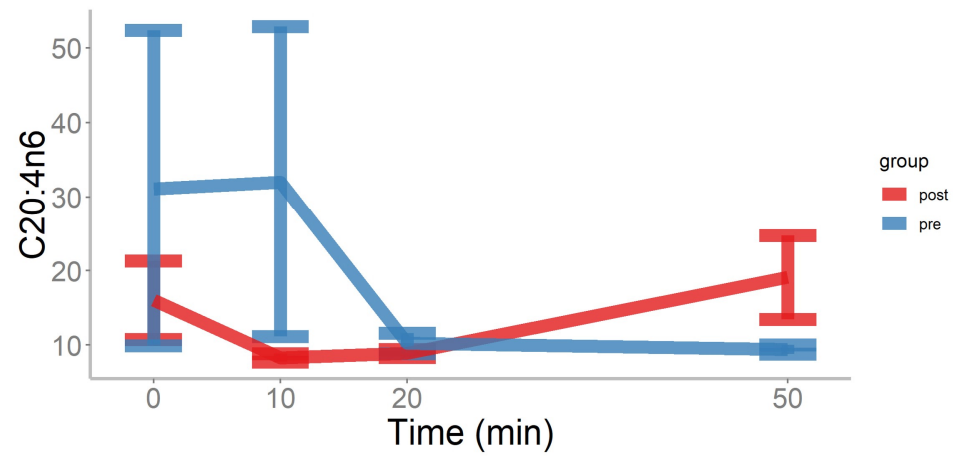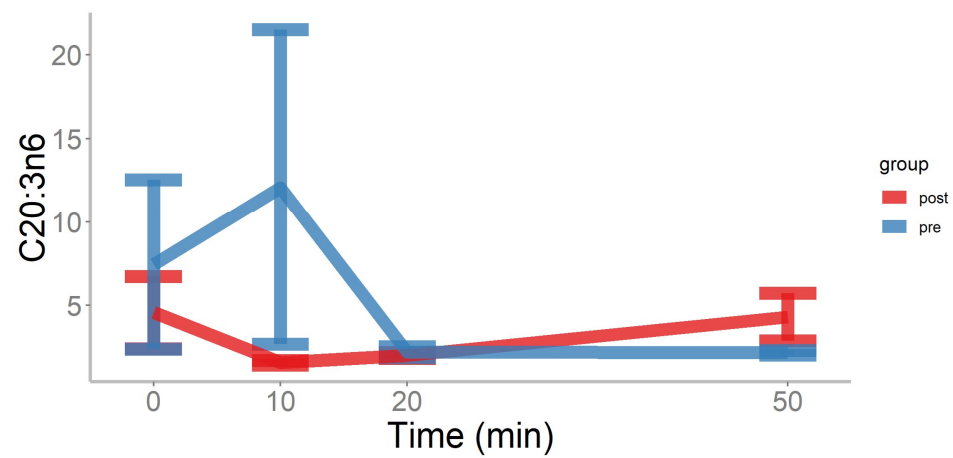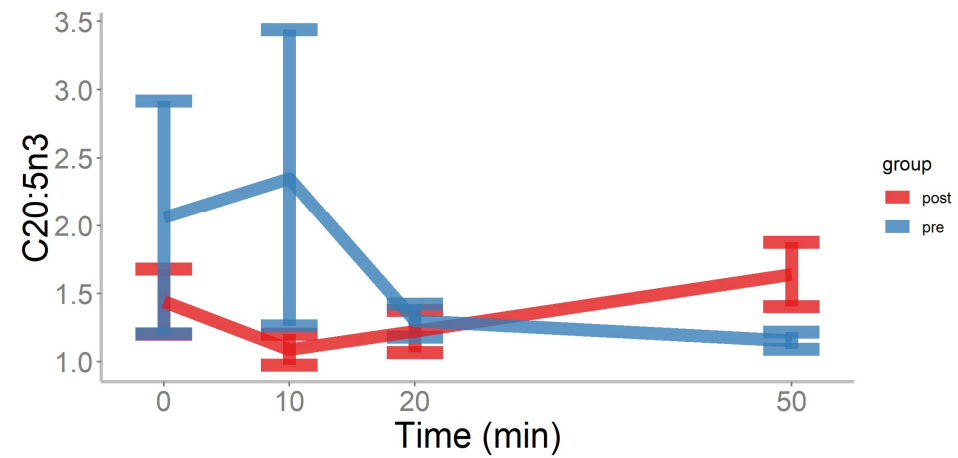

Supplement: Supplementary file 1 — Material S1 [file PHY2-8-e14547-s001.pdf]
